# γδ^+^ T-cell-derived IL-17A stimulates airway epithelial/stromal cells to secrete G-CSF, promoting lung-specific pathogenic Siglec-F^+^ neutrophil development in PPE-induced emphysema

**DOI:** 10.1038/s41423-025-01301-x

**Published:** 2025-06-03

**Authors:** JungHyub Hong, Myeong-Ho Kang, Jinjoo Lee, Min-Suk Cha, Yoe-Sik Bae, Hye Young Kim, Yong Taik Lim, Yong-Soo Bae

**Affiliations:** 1 Department of Biological Sciences, Sungkyunkwan University, Suwon, Republic of Korea; 2 Center for Immune Research on Nonlymphoid Organs, Sungkyunkwan University, Suwon, Republic of Korea; 3 Department of Health Sciences and Technology, Samsung Advanced Institute for Health Sciences and Technology, Sungkyunkwan University, Seoul, Republic of Korea; 4 Laboratory of Mucosal Immunology, Department of Biomedical Sciences, Seoul National University College of Medicine, Seoul, Republic of Korea; 5 Institute of Allergy and Clinical Immunology, Seoul National University Medical Research Center, Seoul, Republic of Korea; 6 Department of Nano Engineering and School of Chemical Engineering, Sungkyunkwan University, Suwon, Republic of Korea

**Keywords:** Porcine pancreatic elastase (PPE), Emphysema, Siglec-F^+^ neutrophils, IL-17A, G-CSF, γδ^+^ T cells, Epithelial/stromal cells, G-CSF receptor signaling, Neutrophils, Chronic inflammation

## Abstract

Neutrophils play a pivotal role in the progression of IL-17-mediated airway inflammation, but the mechanisms underlying their pathological differentiation remain poorly understood. In this study, we identified a distinct population of lung-specific pathogenic Siglec-F^+^ neutrophils in a porcine pancreatic elastase (PPE)-induced mouse model of emphysema. Compared with conventional neutrophils, these Siglec-F^+^ neutrophils exhibited increased phagocytic activity, increased extracellular trap formation, increased production of proinflammatory cytokines, and reduced IL-10 levels. During the early phase of acute inflammation following PPE instillation, IL-17A levels in the lungs increase, which is driven primarily by γδ^+^ T cells. IL-17A stimulated lung epithelial/stromal cells to secrete granulocyte colony-stimulating factor (G-CSF), which promoted the differentiation of Siglec-F^+^ neutrophils via the JAK2/STAT3 pathway and the PI3K-independent mTOR and p38 MAPK signaling pathways. Neutralizing G-CSF or inhibiting JAK2/STAT3, mTOR or p38 MAPK signaling significantly suppressed Siglec-F^+^ neutrophil development, resulting in the alleviation of emphysematous symptoms. Our findings underscore the crucial role of Siglec-F^+^ neutrophils in the pathogenesis of PPE-induced emphysema and suggest that targeting the IL-17A/G-CSF axis or G-CSF receptor downstream signaling pathways may represent a promising therapeutic strategy for treating emphysema.

## Introduction

Chronic obstructive pulmonary disease (COPD) has become the third leading cause of death globally, with both morbidity and mortality expected to increase [1, 2]. COPD is a complex lung condition characterized by chronic inflammation due to exposure to air pollutants or persistent airflow obstruction and is often accompanied by emphysematous changes [3, 4]. In addition to environmental factors, emphysema is significantly associated with genetic risk factors, particularly alpha-1 antitrypsin (AAT) deficiency, which impairs the regulation of neutrophil elastase, leading to destruction of the alveoli and resulting lung damage [5, 6]. Therefore, current treatments for emphysema patients include augmentation therapy, which aims to maintain adequate AAT protein levels throughout their lifetime, delaying lung damage [7].

Interleukin (IL)-17 is a well-known proinflammatory cytokine that induces neutrophilic inflammation through the induction of IL-1β, macrophage inflammatory protein 2, keratinocyte-derived chemokine, and granulocyte colony-stimulating factor (G-CSF), contributing to the host defense reaction against various infectious pathogens [8–10]. In contrast, IL-17 exacerbates the symptoms of autoimmune inflammatory diseases [11–13]. Recently, IL-17, produced by T lymphocytes, has been reported to be associated with pathological processes in asthma, cystic fibrosis, and COPD, where it induces neutrophil-mediated inflammation by stimulating IL-6, tumor necrosis factor-α (TNF-α), G-CSF, and granulocyte‒macrophage colony‒stimulating factor (GM‒CSF) [10, 14, 15]. Although IL-17 is involved in neutrophil recruitment, its role in further functional differentiation of neutrophils remains poorly understood.

Airway neutrophilia is a characteristic feature observed in all COPD patients regardless of age and is associated with the progression of symptoms independent of disease onset. Neutrophils circulate through the blood from the bone marrow and migrate to the lungs, where they perform physiological responses such as degranulation in the inflammatory environment [16]. In the early stages of COPD, the number of neutrophils in the blood increases, resulting in molecular and functional alterations that correlate with a decline in lung function [17]. In COPD patients, G-CSF levels are elevated in bronchoalveolar lavage fluid (BALF), serum, and sputum [18–20]. Consequently, G-CSF has been proposed as a potential therapeutic target and biomarker for COPD. However, the role of G-CSF in COPD remains controversial. On the one hand, G-CSF deficiency has been shown to reduce lung inflammation and damage [18]; on the other hand, administering G-CSF promotes the regeneration of alveolar structures [21]. Therefore, understanding the clear role of G-CSF in each airway inflammatory disease is crucial.

Siglec-F^+^ neutrophils, which express Siglec-F, which is traditionally known as a marker for eosinophils, have recently been identified as a subtype of neutrophils found in inflamed lung, spleen, kidney, heart, and tumor tissues [22–28]. Compared with conventional neutrophils, Siglec-F^+^ neutrophils exhibit an activated phenotype characterized by increased reactive oxygen species (ROS) production and neutrophil extracellular trap (NET) formation, indicating their pathogenic role. Despite this importance, the development of Siglec-F^+^ neutrophils and their role in exacerbating symptoms in emphysema remain unclear.

In this study, we found that (1) a distinct population of lung-resident pathogenic Siglec-F^+^ neutrophils exists in mice with PPE-induced emphysema and that (2) lung-resident γδ^+^ T-cell-derived IL-17A stimulates lung airway epithelial/stromal cells to produce G-CSF, which drives the development of Siglec-F^+^ neutrophils through the JAK2/STAT3, PI3K-independent mTOR, and p38 MAPK signaling pathways, ultimately contributing to the development of emphysema and symptom exacerbation.

## Results

### Siglec-F^+^ neutrophils are newly identified in the lungs of mice with PPE-induced emphysema

**Fig. 1:**
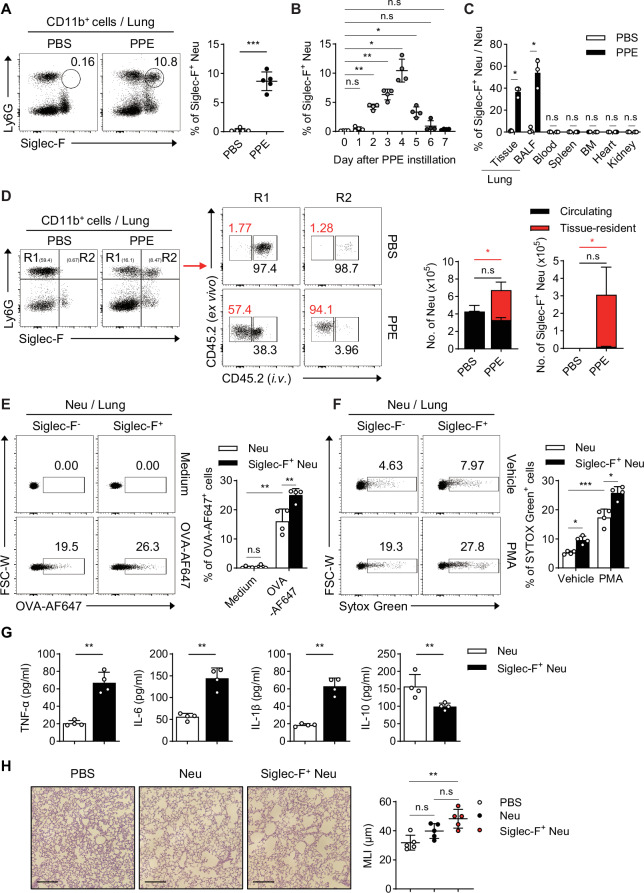
Newly detected Siglec-F^+^ neutrophils in the lungs of PPE-induced emphysema mice. **A** Representative FACS plot showing the CD11b^+^ cell gate and the frequency of Siglec-F^+^ neutrophils in the lungs of PPE-instilled mice on day 4 (*n* = 5 per group). **B** Frequencies of Siglec-F^+^ neutrophils assessed daily over 7 days in the lungs of PPE-instilled mice (*n* = 4 per group). **C** Frequencies of Siglec-F^+^ neutrophils in the organs of PPE-instilled mice on day 4 (*n* = 4 per group). **D** Representative FACS plots showing the CD11b^+^ cell gate in the lungs of PPE-induced emphysema mice assessed 5 min after intravenous staining with anti-CD45-PE. The frequencies of PE^+^ circulating, PE^--^ resident conventional, and Siglec-F^+^ neutrophils were assessed (*n* = 4 per group). **E** Neutrophils harvested from the lungs of PPE-induced emphysema mice on day 4 were cultured with AF647-labeled OVA for 3 h. AF647^+^ cells were then analyzed to assess phagocytic activity (*n* = 4 per group). **F** Neutrophils were isolated from the lungs of PPE-induced emphysema mice on day 4 and then treated with 5 μM PMA for 4 h. NET formation in conventional neutrophils and Siglec-F^+^ neutrophils was assessed by staining with SYTOX Green (*n* = 4 per group). **G** The levels of cytokines secreted by conventional neutrophils and Siglec-F^+^ neutrophils sorted from the lungs of PPE-induced emphysema mice on day 4 were measured 24 h after stimulation with LPS (*n* = 4 per group). **H** Conventional neutrophils and Siglec-F^+^ neutrophils sorted from the lungs of PPE-induced emphysema mice on day 4 were intratracheally transferred into WT mice. Lung tissue histology after H&E staining and the mean linear intercept (MLI) of the recipient mice were then assessed. Scale bar = 200 μm (*n* = 5 per group). Unpaired two-tailed Student’s t test with Welch’s correction **A,** **C,** **G**, unpaired one-way ANOVA with Dunnett’s T3 post hoc test **B,** **H**, and unpaired two-way ANOVA with Tukey’s post hoc test **D–F** were used to measure significance. *P < 0.05, **P < 0.01, ***P < 0.001; *ns*, not significant; error bars indicate the means ± SDs

PPE-induced inflammation and emphysema result in the recruitment of neutrophils [16]. Compared with control mice, mice that were intratracheally instilled with PPE presented an increased mean linear intercept (MLI), indicating the induction of emphysema (Fig. S1A). Unexpectedly, neutrophils expressing Siglec-F were observed in the lungs of mice with PPE-induced emphysema (Figs. 1A, S1B). These Siglec-F^+^ neutrophils exhibited Siglec-E expression similar to that of conventional neutrophils but lacked expression of CCR3, a characteristic marker of eosinophils, confirming their identity as neutrophils (Fig. S1C). The presence of Siglec-F^+^ neutrophils peaked 4 days after PPE instillation and nearly disappeared on day 7 (Figs. 1B, S1D). Several studies have shown the presence of Siglec-F^+^ neutrophils in various organs [22–28]. In our study, these cells were exclusively observed in the lung tissue and BALF (Figs. 1C, S1E). In an intravenous staining experiment in which a fluorescent-conjugated CD45.2 Ab was used to track the circulating cells, the Siglec-F^+^ neutrophils were not stained, although most conventional neutrophils were stained (Fig. 1D). This finding suggests that, unlike the majority of conventional neutrophils, Siglec-F^+^ neutrophils reside in the lung tissue. Notably, Siglec-F^+^ neutrophils displayed greater phagocytic activity (Fig. 1E) and NET levels than conventional neutrophils did (Fig. 1F). Additionally, they produced elevated levels of proinflammatory cytokines such as TNF-α, IL-6, and IL-1β, while the level of the anti-inflammatory cytokine IL-10 was reduced (Fig. 1G). Furthermore, when Siglec-F^+^ neutrophils isolated from the lungs of PPE-induced emphysema mice were transferred intratracheally (*i.t*.) into naïve wild-type (WT) mice, the lungs of the recipient mice presented a greater increase in the MLI than did those of conventional neutrophils or phosphate-buffered saline (PBS)-treated control mice (Fig. 1H). Together, these findings suggest that Siglec-F^+^ neutrophils represent a distinct population in the PPE-induced emphysema model and play a more pathogenic role than do conventional neutrophils by residing specifically in the lung and exacerbating emphysema.

### IL-17A is essential for the development of Siglec-F^+^ neutrophils in PPE-induced emphysema

**Fig. 2:**
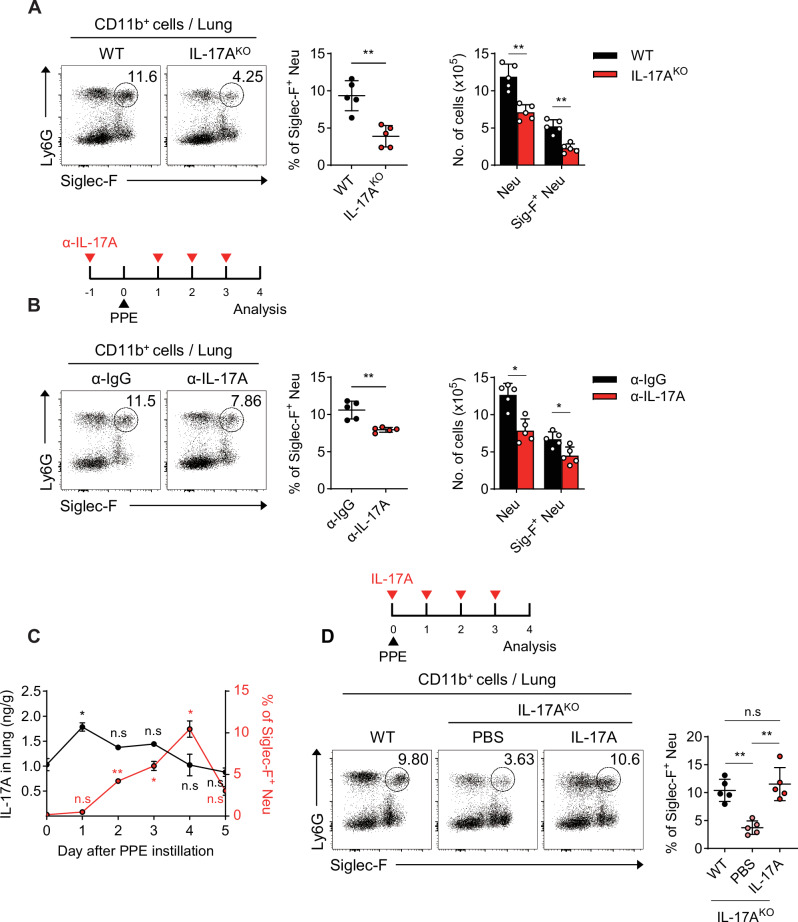
IL-17A is essential for the development of Siglec-F^+^ neutrophils in the lungs of PPE-induced emphysema mice. **A** Representative FACS plot of the CD11b^+^ cell gate and the frequency and absolute cell number of Siglec-F^+^ neutrophils in the lungs of WT and IL-17A^KO^ mice 4 days after PPE instillation (*n* = 4 per group). **B** Experimental protocol for anti-IL-17A administration and PPE instillation. Representative FACS plot of the CD11b^+^ cell gate and the frequency and absolute cell number of Siglec-F^+^ neutrophils in the lungs of PPE-induced emphysema mice in which IL-17A was depleted (*n* = 5 per group). **C** The concentrations of IL-17A (black circles) and the frequencies of Siglec-F^+^ neutrophils (red circles) were assessed over 5 days following PPE instillation (*n* = 4 per group). **D** Experimental protocol for recombinant IL-17A administration and PPE instillation. The frequency of Siglec-F^+^ neutrophils in the lungs of WT and IL-17A^KO^ mice was assessed 4 days after PPE instillation, with or without recombinant IL-17A administration to IL-17A^KO^ mice (*n* = 5 per group). Unpaired two-tailed Student’s *t* test with Welch’s correction **A,** **B**, unpaired one-way ANOVA with Dunnett’s T3 post hoc test **C**, and unpaired two-way ANOVA with Tukey’s post hoc test **D** were used to measure significance. *P < 0.05, **P < 0.01; *ns*, not significant; error bars indicate the mean ± SD (SEM in C)

IL-17A plays a significant role in the development of PPE-induced emphysema, and its deficiency has been shown to delay disease progression [29]. However, the relationship between IL-17A and Siglec-F^+^ neutrophils in emphysema is not well understood. To explore this association, we induced emphysema in IL-17A-knockout (IL-17A^KO^) mice and examined changes in the population of Siglec-F^+^ neutrophils. The population and number of Siglec-F^+^ neutrophils were lower in the IL-17A-deficient mice than in the WT mice (Fig. 2A). Additionally, lung histology revealed a lower MLI in IL-17A-deficient mice than in WT mice (Fig. S2A), which is consistent with a previous report [29]. Neutralizing IL-17A also reduced the number of Siglec-F^+^ neutrophils in emphysema-induced mice, mirroring the effect observed in IL-17A-deficient mice (Fig. 2B). Similarly, IL-17A-neutralized mice presented a lower MLI than IgG controls did (Fig. S2B). To assess the potential involvement of IL-17F in the PPE-induced emphysema model, we used an anti-IL-17F neutralizing antibody. The results revealed a decreasing trend in the Siglec-F⁺ neutrophil population, although the reduction was less pronounced than that observed with anti–IL-17A antibody treatment (Fig. S2C). These findings are consistent with a previous report indicating that IL-17A is more potent than IL-17F in promoting neutrophil-mediated airway inflammation [30]. To further investigate the correlation between IL-17A and Siglec-F^+^ neutrophils during emphysema progression, we examined the kinetics of Siglec-F^+^ neutrophil frequency and the IL-17A concentration. Following PPE installation, IL-17A levels increased rapidly within a day and then gradually declined, whereas the frequency of Siglec-F^+^ neutrophils increased from day 2 to day 4 after emphysema onset and subsequently declined (Fig. 2C). Moreover, the administration of recombinant IL-17A restored the reduced Siglec-F^+^ neutrophil population in the IL-17A-deficient mice to levels comparable to those in the WT mice (Fig. 2D). These findings suggest a positive correlation between the IL-17A concentration and the frequency of Siglec-F^+^ neutrophils in patients with PPE-induced emphysema.

### Lung γδ^+^ T cells are the major source of IL-17A in the PPE-induced emphysema model

**Fig. 3:**
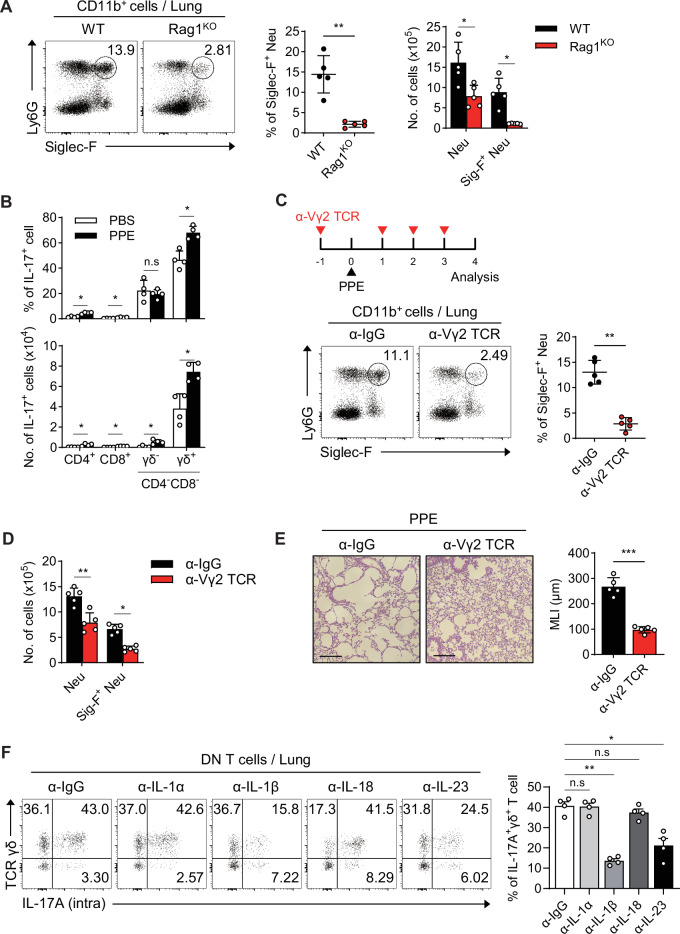
Lung γδ^+^ T cells are the major source of IL-17A in the lungs of PPE-induced emphysema mice. **A** The frequency and quantification of Siglec-F^+^ neutrophils in the lungs of WT and Rag1^KO^ mice 4 days after PPE instillation (*n* = 5 per group). **B** The frequency and absolute number of IL-17A-expressing cells among T-cell subsets in the lungs of WT mice were analyzed on day 2 after PPE instillation (*n* = 4 per group). **C** Experimental protocol for anti-Vγ2 TCR administration in the PPE-induced emphysema model. The frequency of Siglec-F^+^ neutrophils in the lungs of PPE-induced emphysema mice was assessed on day 4 after depletion of γδ^+^ T cells (*n* = 5 per group). **D** Absolute number of Siglec-F^+^ neutrophils in the lungs of PPE-induced emphysema mice on day 4 after depletion of γδ^+^ T cells (*n* = 5 per group). **E** Lu*n*g tissue histology and the MLI on day 4 in PPE-instilled WT mice pretreated with either anti-IgG or anti-Vγ2 antibodies. Scale bar = 200 μm (*n* = 5 per group). **F** The frequency of γδ^+^ T cells in the lungs of PPE-induced emphysema mice two days after the administration of cytokine-blocking antibodies (*n* = 4 per group). Unpaired two-tailed Student’s t test with Welch’s correction **A left**-**C, and E**, unpaired one-way ANOVA with Dunnett’s T3 post hoc test **F**, and unpaired two-way ANOVA with Tukey’s post hoc test **A right,** **D** were used to measure significance. *P < 0.05, **P < 0.01, ***P < 0.001; *ns*, not significant; error bars indicate the means ± SDs

We induced emphysema in Rag1-knockout (Rag1^KO^) mice and examined changes in the Siglec-F^+^ neutrophil population. The results revealed that the number of Siglec-F^+^ neutrophils was significantly lower in Rag1^KO^ mice than in WT mice (Fig. 3A). IL-17A is produced by various T-cell subsets, including Th17, Tc17, and double-negative T cells [29, 31, 32], as well as type 3 innate lymphoid cells (ILC3s) [33]. In our study, γδ^+^ T cells were the prominent IL-17A-expressing cells, with the highest absolute cell count among the T-cell subsets (Figs. 3B, S3A). Although IL-17A was also expressed by ILC3s in emphysema, the frequency and absolute cell number of IL-17A^+^ ILC3s were markedly lower than those of γδ^+^ T cells (Fig. S3B). Therefore, we postulated that γδ^+^ T-cell-derived IL-17A may be linked to the Siglec-F^+^ neutrophil population. Indeed, treatment with an anti-Vγ2 TCR antibody significantly reduced the frequency (Fig. 3C) and absolute cell number (Fig. 3D) of the Siglec-F^+^ neutrophils in the lungs. When γδ^+^ T cells were depleted in PPE-treated mice, disease symptoms were significantly alleviated (Fig. 3E). Cytokine-blocking assays revealed that IL-1β and IL-23 play major roles in stimulating γδ^+^ T cells to produce IL-17A (Fig. 3F). These findings suggest that lung γδ^+^ T cells, which are activated by inflammatory cytokines, are the major source of IL-17A in PPE-induced emphysema and are closely associated with the development of Siglec-F^+^ neutrophils.

### IL-17A-derived G-CSF plays a crucial role in the development of Siglec-F^+^ neutrophils in the lungs of emphysema mice

**Fig. 4:**
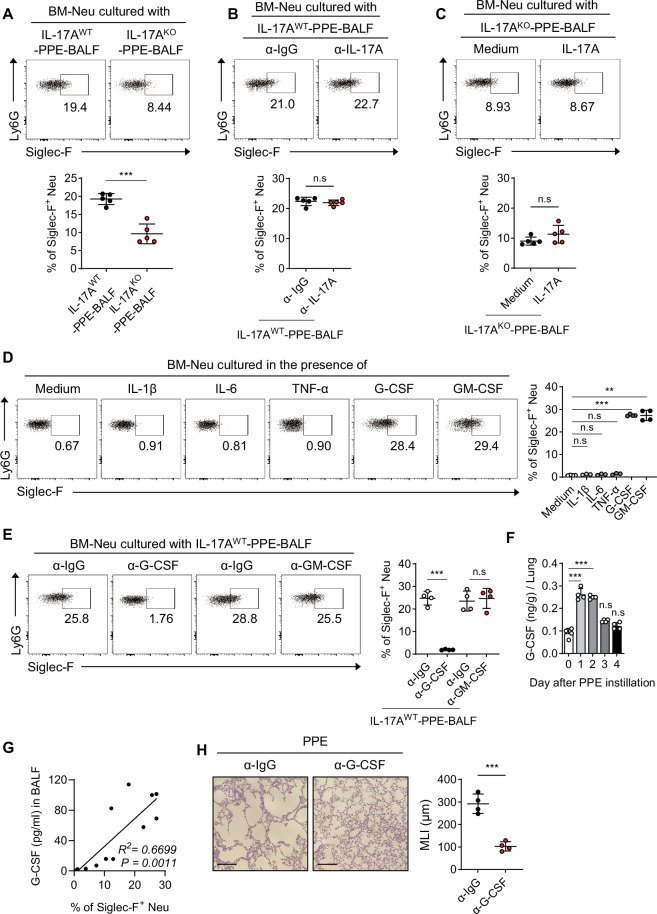
IL-17A-derived G-CSF plays a crucial role in the development of Siglec-F^+^ neutrophils in the lungs of emphysema mice. **A** The frequency of Siglec-F^+^ neutrophils was assessed in BM-neutrophil cultures after 48 h of incubation with cell-free BALF isolated from PPE-instilled WT and IL-17A^KO^ mice (*n* = 5 per group). **B** The frequency of Siglec-F^+^ neutrophils in BM-neutrophil cultures after 48 h of incubation with cell-free BALF isolated from PPE-instilled WT mice in the presence of either anti-IgG or anti-IL-17A (*n* = 5 per group). **C** The frequency of Siglec-F^+^ neutrophils in BM-neutrophil cultures after 48 h of incubation with cell-free BALF isolated from PPE-instilled IL-17A^KO^ mice in the presence of recombinant IL-17A (*n* = 5 per group). **D** The frequency of Siglec-F^+^ neutrophils in BM-neutrophil cultures after 48 h of incubation with recombinant cytokines (*n* = 4 per group). **E** The frequency of Siglec-F^+^ neutrophils in BM-neutrophil cultures after 48 h of incubation with cell-free BALF isolated from PPE-instilled WT mice, along with blocking antibodies for each cytokine (*n* = 4 per group). **F** G-CSF concentrations in the lungs of PPE-instilled mice were assessed daily (*n* = 4 per group). **G** Pearson’s correlation was used to assess the frequency of Siglec-F^+^ neutrophils in BM-neutrophil cultures with cell-free BALF and the concentration of G-CSF in BALF from PBS- or PPE-instilled mice (*n* = 12). **H** WT mice were *i.t*. administered either anti-G-CSF antibody or anti-IgG before PPE instillation. Lung tissue histology after H&E staining and MLI assessment was conducted on day 4. Scale bar = 200 μm (*n* = 4 per group). Unpaired two-tailed Student’s t test with Welch’s correction **A,** **B,** **C**, and **H** and unpaired one-way ANOVA with Dunnett’s T3 post hoc test (**D,** **E**, and **F**) were used to measure significance. **P < 0.01, ***P < 0.001; *n*s, not significant; error bars indicate the mean ± SD

Neutrophils are affected by BALF, which contains pulmonary environmental factors that reduce neutrophil apoptosis and extend their lifespan [34]. We hypothesized that the altered lung environment in emphysema includes factors that induce the differentiation of neutrophils into Siglec-F^+^ neutrophils. Notably, BALF from PPE-treated mice, unlike that from PPE-untreated control mice, could induce the differentiation of bone marrow (BM)-derived neutrophils into Siglec-F^+^ neutrophils (Fig. S4A). Furthermore, this differentiation into Siglec F^+^ neutrophils decreased as the dilution rate of PPE-BALF increased (Fig. S4B). These findings suggest that soluble factors in the PPE-induced lung environment drive the differentiation of pathogenic Siglec-F^+^ neutrophils. To explore whether IL-17A influences the differentiation of Siglec-F^+^ neutrophils, we isolated BALF from PPE-treated IL-17A^WT^ and IL-17A^KO^ mice and then cultured BM-neutrophils with these BALF samples. Compared with that in IL-17AWT mice, the differentiation of Siglec-F^+^ neutrophils in BALF from IL-17A^KO^ mice was reduced (Fig. 4A). However, neutralizing IL-17A in IL-17A^WT^-PPE-BALF did not affect Siglec-F^+^ neutrophil differentiation (Fig. 4B), and adding recombinant IL-17A to IL-17A^KO^-PPE-BALF did not increase the differentiation of Siglec-F^+^ neutrophils (Fig. 4C). These results indicate that IL-17A is essential but does not directly affect Siglec-F^+^ neutrophil differentiation. IL-17 stimulates lung airway epithelial/stromal cells to produce CXCL1, a potent neutrophil chemoattractant [30, 35]. When CXCL1 was blocked in PPE-induced emphysema mice, neutrophil migration into the lungs was markedly reduced, and the number of Siglec-F⁺ neutrophils was significantly decreased (Fig. S4C middle). However, the frequency of neutrophils differentiating into a Siglec-F⁺ subset remained unchanged (Fig. S4C left and right). These results suggest that IL-17A-derived CXCL1 is not directly involved in the differentiation of lung-migrated neutrophils into Siglec-F⁺ neutrophils. It is also well known that IL-17A activates other cells, leading to the secretion of proinflammatory cytokines such as IL-1β, IL-6, TNF-α, G-CSF, and GM-CSF [36]. When cultured with these inflammatory cytokines, only G-CSF and GM-CSF induced differentiation into Siglec-F^+^ neutrophils, whereas the other cytokines did not (Fig. 4D). Neutralizing G-CSF in IL-17A^WT^-PPE-BALF significantly inhibited the differentiation of Siglec-F^+^ neutrophils, whereas neutralizing GM-CSF had no effect (Fig. 4E). In PPE-induced emphysema, G-CSF levels increased until day 2 and then decreased (Fig. 4F), whereas GM-CSF levels showed little change compared with those in the control (Fig. S4D). There was a positive correlation between the concentration of G-CSF in the BALF and the increase in the Siglec-F^+^ neutrophil population (Fig. 4G). In addition, as the concentration of G-CSF in BM-neutrophil cultures increased, Siglec-F^+^ neutrophil differentiation increased in a dose-dependent manner (Fig. S4E). Compared with the control condition, neutralizing G-CSF in PPE-induced emphysema mice reduced alveolar damage (Fig. 4H) and decreased the number of Siglec-F^+^ neutrophils and the cell count (Fig. S4F). Although BM-neutrophils cultured with IL-17A^WT^-PPE-BALF differentiated into Siglec-F^+^ neutrophils, BM-eosinophils did not (Fig. S4G). Similarly, BM-neutrophils cultured with G-CSF differentiated into Siglec-F^+^ neutrophils, whereas BM-eosinophils did not (Fig. S4H). Like BM-derived neutrophils, lung-derived neutrophils also efficiently differentiated into Siglec-F^+^ neutrophils in response to G-CSF (Fig. S4I). Collectively, these findings suggest that γδ^+^ T-cell-derived IL-17A does not directly induce Siglec-F^+^ neutrophil differentiation. Instead, it seems to stimulate other cells to secrete G-CSF, which is crucial for the development of Siglec-F^+^ neutrophils.

### Lung epithelial/stromal cells secrete G-CSF when stimulated with γδ^+^ T-cell-derived IL-17A

**Fig. 5:**
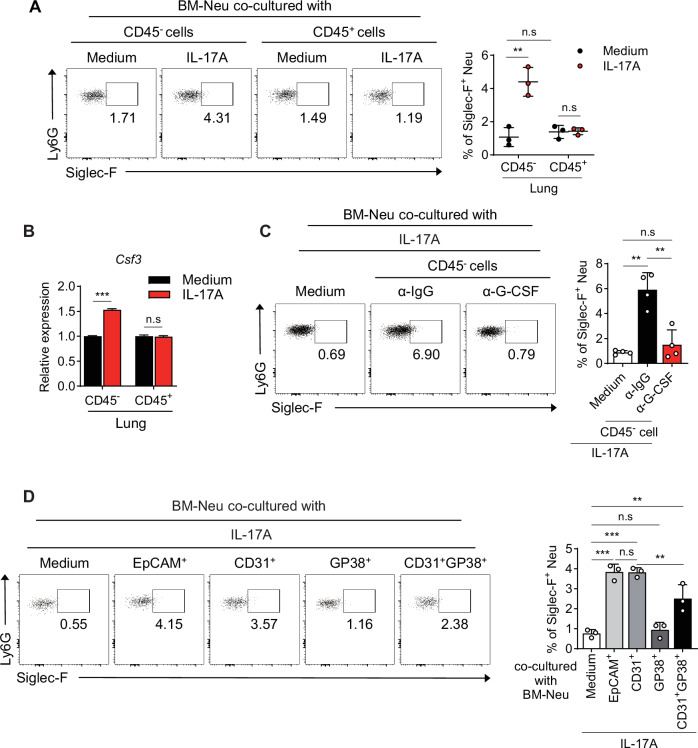
Lung epithelial/stromal cells secrete G-CSF when stimulated with IL-17A. **A** BM-derived neutrophils were cocultured for 48 h with lung CD45^-^ nonimmune cells or with CD45^+^ immune cells, with or without recombinant IL-17A. The frequency of Siglec-F^+^ neutrophils was assessed in each culture (*n* = 3 per group). **B** mRNA levels of *Csf3* in lung CD45^-^ nonimmune cells and CD45^+^ immune cells measured by qRT‒PCR after stimulation with recombinant IL-17A for 6 h (*n* = 5 per group). **C** BM-derived neutrophils were cocultured for 48 h with lung CD45^-^ nonimmune cells in the presence of anti-G-CSF antibody or anti-IgG, and the frequency of Siglec-F^+^ neutrophils was assessed in each culture (*n* = 4 per group). **D** BM-derived neutrophils were cocultured with various types of nonimmune cells sorted from the lungs of WT mice in the presence of recombinant IL-17A. The frequency of Siglec-F^+^ neutrophils was assessed in each culture (*n* = 3 per group). Unpaired two-tailed Student’s t test with Welch’s correction **B**, unpaired one-way ANOVA with Dunnett’s T3 post hoc test **D** and unpaired two-way ANOVA with Tukey’s post hoc test **A,** **C** were used to measure significance. *P < 0.05, **P < 0.01, ***P < 0.001; *ns*, not significant; error bars indicate the means ± SDs

Next, we investigated the source of G-CSF, which is essential for the development of Siglec-F^+^ neutrophils in the presence of γδ^+^ T-cell-derived IL-17A. BM-derived neutrophils were cocultured with lung-derived CD45^-^ nonimmune cells or CD45^+^ immune cells in the presence of IL-17A. When cocultured with IL-17A-stimulated CD45^-^ nonimmune cells, BM neutrophils differentiated into Siglec-F^+^ neutrophils, whereas those cocultured with 17A-stimulated CD45^+^ immune cells did not (Fig. 5A). These findings suggest that nonimmune cells, rather than immune cells, are likely the source of G-CSF when stimulated by IL-17A. Additionally, IL-17A stimulation significantly increased the mRNA levels of G-CSF in nonimmune cells, whereas no changes were observed in CD45⁺ immune cells under the same conditions. (Figs. 5B, S5). Furthermore, the development of Siglec-F^+^ neutrophils from BM neutrophils cocultured with nonimmune cells under IL-17A stimulation was significantly inhibited by G-CSF neutralization (Fig. 5C). These findings suggest that IL-17A-activated nonimmune cells are the source of G-CSF, which is responsible for the development of Siglec-F^+^ neutrophils. In the lungs, various nonimmune cells are involved in lung inflammation [37]. To identify the specific nonimmune cells responsible for Siglec-F^+^ neutrophil differentiation when stimulated with IL-17A, BM neutrophils were cocultured with epithelial EpCAM^+^ cells, blood endothelial cells (CD31^+^), lymphatic endothelial cells (CD31^+^GP38^+^), or fibroblasts (GP38^+^) from WT lungs in the presence of IL-17A. Among these, the differentiation of Siglec-F^+^ neutrophils was most prominent in cocultures with EpCAM^+^ epithelial cells and CD31^+^ blood endothelial cells (Fig. 5D). Overall, our findings indicate that lung EpCAM^+^ epithelial cells and CD31^+^ blood endothelial cells are the major sources of G-CSF in lung epithelial/stromal cells when stimulated with IL-17A, which plays a pivotal role in the development of Siglec-F^+^ neutrophils in PPE-induced emphysema mice.

### G-CSF-stimulated neutrophils exhibited pathogenic gene expression profiles through G-CSFR downstream signaling pathways

**Fig. 6:**
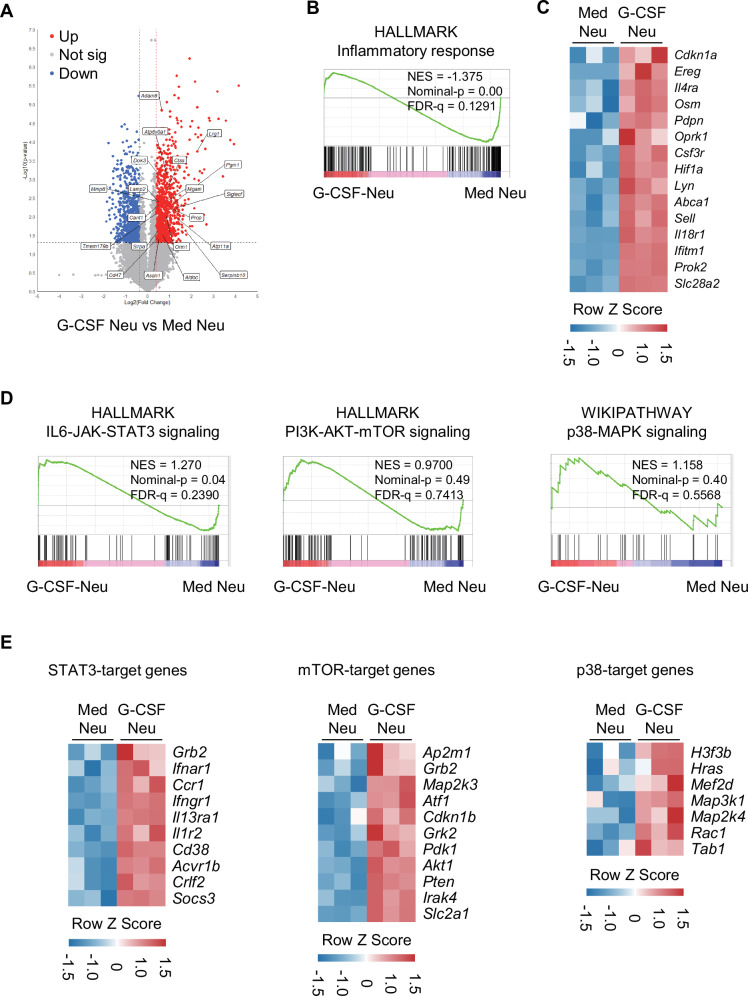
RNA bulk-seq analysis of G-CSF-stimulated neutrophils versus control neutrophils. **A** Volcano plot of differential gene expression between G-CSF-stimulated (6 h) BM-neutrophils (G-CSF neutrophils) and medium-cultured control BM-neutrophils (med neutrophils). **B** Gene set enrichment analysis (GSEA) of gene sets associated with inflammatory responses in G-CSF-stimulated BM-neutrophils compared with control BM-neutrophils cultured in medium. **C** Heatmap of inflammatory response-related genes from RNA bulk-seq analysis of G-CSF-stimulated BM-neutrophils compared with control BM-neutrophils. **D** GSEA of gene sets associated with hallmark IL6-JAK-STAT3 signaling (left), hallmark PI3K-AKT-mTOR signaling (middle), and the BIOCARTA p38 MAPK pathway (right) in G-CSF-stimulated BM-neutrophils compared with control BM-neutrophils. **E** Heatmap of STAT3 target genes, mTOR target genes, and p38 target genes from RNA bulk-seq analysis of G-CSF-stimulated BM-neutrophils compared with control BM-neutrophils

Mature neutrophils become activated and release granules, contributing to host defense against infection but sometimes causing damage to host tissue, such as lung injury, in patients with COPD [38, 39]. Volcano plots following bulk-sequencing analysis revealed that many genes associated with the expression of tertiary granules were upregulated in G-CSF-stimulated BM-neutrophils compared with control BM-neutrophils (Fig. 6A). Furthermore, gene set expression analysis revealed a marked increase in inflammatory response-related gene sets (Fig. 6B) and inflammation-associated genes (Fig. 6C). These results provide evidence for the pathogenic characteristics of Siglec-F^+^ neutrophils generated in the presence of G-CSF. Next, we conducted gene set enrichment analysis (GSEA) to identify the signaling pathways involved in the differentiation of Siglec-F^+^ neutrophils downstream of G-CSF receptor (G-CSFR) signaling. We found significant enrichment of genes involved in the JAK-STAT3, PI3K-mTOR, and p38 MAPK pathways (Fig. 6D). Additionally, the expression levels of the target genes associated with STAT3, mTOR, and p38 were notably increased in G-CSF-stimulated BM-neutrophils (Fig. 6E). These findings suggest that the downstream signaling pathways of G-CSFRs, specifically JAK-STAT, PI3K-mTOR, and p38-MAPK, and their target genes are involved in the differentiation of BM-derived neutrophils into Siglec-F^+^ neutrophils. This differentiation driven by G-CSF secreted from IL-17-stimulated lung epithelial/stromal cells plays a crucial role in the pathogenesis of PPE-induced emphysema.

### G-CSF receptor downstream signaling pathways are implicated in the expression of Siglec-F in neutrophils

**Fig. 7:**
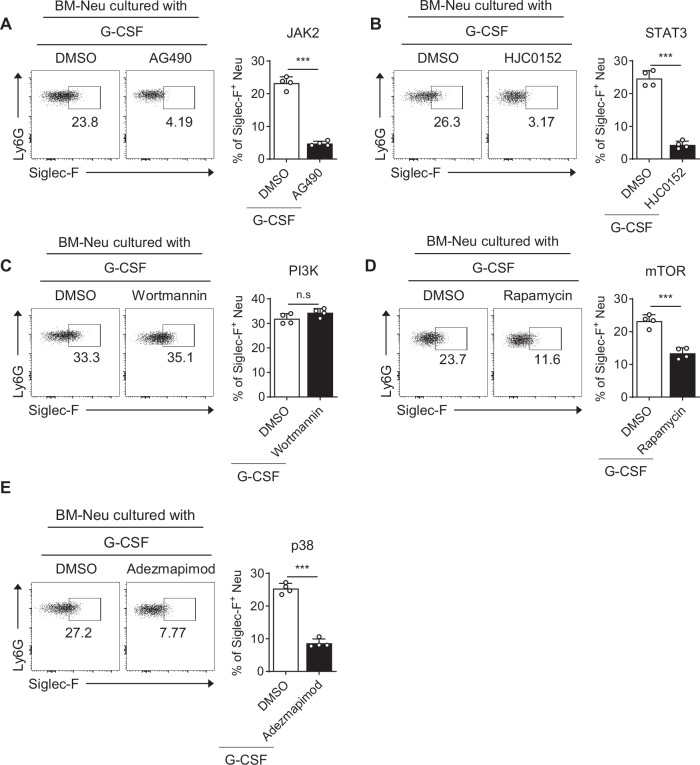
Signaling pathways downstream of the G-CSF receptor involved in the development of Siglec-F^+^ neutrophils. The frequency of Siglec-F^+^ neutrophils was assessed in BM-neutrophil cultures after 48 h of incubation with recombinant G-CSF in the presence of AG490 (JAK2 inhibitor) **A**, HJC0152 (STAT3 inhibitor) **B**, wortmannin (PI3K inhibitor) **C**, rapamycin (mTOR inhibitor) **D**, or adezmapimod (p38 inhibitor) **E**, *n* = 4 per group in each experiment. Unpaired two-tailed Student’s t test with Welch’s correction was used to measure significance. ***P < 0.001; *ns* not significant; error bars indicate the mean ± SD

We then investigated whether the potential intracellular signaling pathways downstream of G-CSFR predicted in silico are specifically involved in the differentiation of Siglec-F^+^ neutrophils. The JAK/STAT pathway is a well-established signaling cascade activated by G-CSF [40, 41]. Treatment with the JAK2 inhibitor AG490 significantly attenuated the G-CSF-mediated differentiation of Siglec-F^+^ neutrophils (Figs. 7A, S6A). STAT3, a downstream target of JAK2, is involved in neutrophil differentiation during granulopoiesis [42]. Similarly, the inhibition of STAT3 phosphorylation by the STAT3 inhibitor HJC0152 also reduced the G-CSF-induced differentiation of Siglec-F^+^ neutrophils (Figs. 7B, S6B). Although G-CSF activates PI3K, leading to the phosphorylation of mTOR [43], treatment with the PI3K inhibitor wortmannin did not affect the differentiation of Siglec-F^+^ neutrophils (Figs. 7C, S6C). However, the mTOR inhibitor rapamycin reduced G-CSF-induced Siglec-F^+^ neutrophil differentiation (Figs. 7D, S6D), indicating that PI3K-independent mTOR plays a partial role in this process. Furthermore, the p38 inhibitor adezmapimod significantly inhibited the differentiation of G-CSF-induced Siglec-F^+^ neutrophils (Figs. 7E, S6E). STAT5 is another key signaling molecule downstream of G-CSFR that promotes neutrophil proliferation and survival [44]. However, the inhibition of STAT5 phosphorylation only mildly attenuated the G-CSF-mediated differentiation of Siglec-F^+^ neutrophils (Fig. S6F). These findings suggest that the differentiation of Siglec-F^+^ neutrophils induced by G-CSF primarily depends on the JAK2/STAT3 axis and the PI3K-independent mTOR and p38 MAPK pathways.

### Direct depletion of Siglec-F^+^ neutrophils or blockade of G-CSFR signaling alleviates emphysema symptoms

**Fig. 8:**
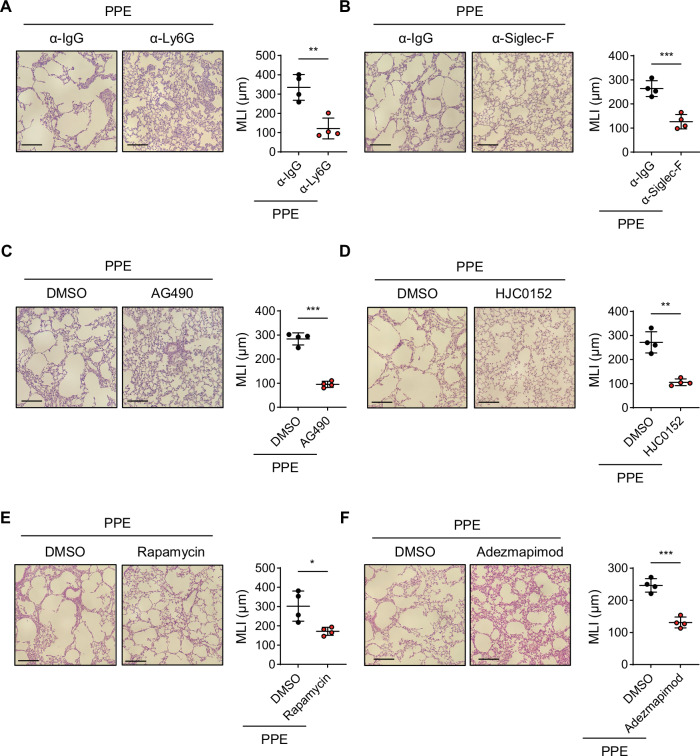
Depletion of Siglec-F^+^ neutrophils or inhibition of G-CSFR signaling alleviated emphysema symptoms. WT mice were treated *i.t*. with anti-Ly6G antibody **A** or anti-Siglec-F antibody **B** before instillation with PPE. Lung tissue histology after H&E staining and MLI assessment were conducted 4 days after PPE instillation. Scale bar = 200 μm (*n* = 4 per group in each experiment). PPE-instilled mice were treated *i.t*. with AG490 (JAK2 inhibitor) **C**, HJC0152 (STAT3 inhibitor) **D**, rapamycin (mTOR inhibitor) **E**, or Adezmapimod (p38 inhibitor) **F**. Lung tissue histology after H&E staining and MLI assessment were conducted 4 days after PPE instillation. Scale bar = 200 μm (*n* = 4 per group). Unpaired two-tailed Student’s t test with Welch’s correction was used to measure significance. *P < 0.05, **P < 0.01, ***P < 0.001; the error bars indicate the means ± SDs

To investigate whether targeting Siglec-F^+^ neutrophils could alleviate the symptoms of emphysema, depletion antibodies or signaling blockades were administered daily following the induction of emphysema. *I.t*. administration of anti-Ly6G or anti-Siglec-F effectively depleted the population of Siglec-F^+^ neutrophils (Fig. S7A, B). Compared with control mice treated with anti-IgG, mice treated with anti-Ly6G and anti-Siglec-F antibodies presented a significant reduction in the MLI (Fig. 8A, B). Eosinophils also contribute to symptom exacerbation in an emphysema model [45]. Although CD11b^+^Ly6G^-^Siglec-F^+^ eosinophil levels remained unaffected even when Ly6G^+^ cells were completely depleted (Fig. S7A), emphysema symptoms were significantly alleviated (Fig. 8A), highlighting the crucial role of Siglec-F^+^ neutrophils in the exacerbation of emphysema. The reduction in MLI was more pronounced with Ly6G depletion (Fig. 8A) than with direct targeting of Siglec-F^+^ neutrophils (Fig. 8B), suggesting that blocking the differentiation of neutrophils into Siglec-F^+^ neutrophils may be more efficient than depleting established pathogenic Siglec-F^+^ neutrophils in controlling emphysema symptoms. Given that anti-Siglec-F antibodies can deplete alveolar macrophages (AMs), potentially contributing to disease alleviation, we employed clodronate to assess the effect of AM depletion on disease severity. Clodronate treatment effectively eliminated AMs (Fig. S7C), whereas Siglec-F⁺ neutrophils exhibited only a slight reduction in number and frequency, likely due to their phagocytic activity (Fig. S7D, E). Notably, clodronate treatment resulted in only a modest decrease in the disease score (Fig. S7F). These findings suggest that the amelioration of emphysema symptoms observed following Siglec-F⁺ cell depletion is unlikely to be due to AM depletion but is instead driven primarily by the removal of pathogenic Siglec-F⁺ neutrophils. We next explored whether blocking the signaling pathways involved in the differentiation of Siglec-F^+^ neutrophils could effectively control emphysema symptoms in PPE-treated mice. *I.t*. administration of AG490 (JAK2 inhibitor) or HJC0152 (STAT3 inhibitor) significantly reduced both the frequency and absolute cell number of Siglec-F^+^ neutrophils in the lungs of PPE-treated mice (Fig. S8A, B), and this reduction was accompanied by marked alleviation of emphysema symptoms, as assessed by MLI (Fig. 8C, D). Notably, treatment with the STAT3 inhibitor HJC0152 also led to a substantial reduction in conventional neutrophils (Fig. S8B). This finding likely reflects the broader role of STAT3 in both Siglec-F⁺ neutrophil differentiation and IL-17 expression. Thus, STAT3 inhibition may have suppressed the IL-17/CXCL1 axis, thereby impairing overall neutrophil migration into the lung. In contrast, *i.t*. administration of rapamycin (mTOR inhibitor) and Adezmapimod (a p38 inhibitor) also reduced the frequency and absolute cell number of Siglec-F^+^ neutrophils without affecting the populations of conventional neutrophils in the lungs (Fig. S8C, D) and significantly improved emphysema pathology compared with that in DMSO-treated control mice (Fig. 8E, F). Our findings suggest that direct targeting of Siglec-F^+^ neutrophils with a depleting antibody or G-CSF signaling inhibitor other than a STAT3 inhibitor for blocking the development of Siglec-F^+^ neutrophils could be a potential therapeutic approach for emphysema.

## Discussion

During airway inflammation, neutrophils migrate persistently into the lungs, where they accumulate and release granules and vesicles that impact tissue remodeling and degradation, promoting lung destruction. However, the pulmonary environmental factors influencing the differentiation of neutrophils with pathogenic properties in airway inflammation remain poorly understood. In an emphysema model characterized by neutrophilia, we identified a specific neutrophil subtype that expresses Siglec-F on the surface. This study aimed to elucidate the detailed mechanisms underlying the differentiation of Siglec-F^+^ neutrophils and their comprehensive pathophysiological properties.

In the emphysema model, neutrophilia is caused primarily by the migration and accumulation of neutrophils in the lung [16]. However, this neutrophil population is highly heterogeneous, and we identified a novel Siglec-F^+^ subset in the lungs of emphysema mice. While conventional neutrophils circulate throughout the body, Siglec-F^+^ neutrophils are exclusively found in lung tissues and BALF, indicating their tissue-specific residency. Neutrophils typically traverse inflamed tissue and undergo transendothelial migration in response to host-derived cytokines [46, 47]. Our findings demonstrate that in the PPE-induced emphysema model, lung neutrophils recruited from the bloodstream further differentiate into Siglec-F^+^ neutrophils, eventually acquiring lung-specific residency. Compared with conventional neutrophils, Siglec-F^+^ neutrophils display a more pathogenic phenotype characterized by increased phagocytic activity, increased levels of NETs, increased production of proinflammatory cytokines such as TNF-α, IL-6, and IL-1β, and decreased production of the anti-inflammatory cytokine IL-10.

The lungs create a unique pulmonary environment with organ-specific immune cells such as resident dendritic cells and alveolar macrophages [48]. We have demonstrated that lung-resident neutrophils have distinct gene expression profiles that distinguish them from bone marrow and blood neutrophils, highlighting the impact of the unique environment of the lungs [34]. In early-stage COPD, molecular and functional changes in neutrophils have been demonstrated to be associated with impaired lung function [17]. However, the soluble factors and detailed immunological mechanisms driving further differentiation of neutrophils in emphysema models remain unknown. Our findings showed that IL-17A^WT^-PPE-BALF was more effective than IL-17A^KO^-PPE-BALF in promoting the differentiation of Siglec-F^+^ neutrophils, suggesting a role for IL-17A in this process. However, neither IL-17A neutralization nor the addition of recombinant IL-17A affected Siglec-F^+^ neutrophil differentiation in the PPE-BALF experiments, indicating that IL-17A is not directly involved in this differentiation.

The lung, as a barrier that interacts with the external environment through the airways, provides a supportive habitat for resident immune cells to control infections. However, excessive inflammatory responses can alter the resident immune cells in nonlymphoid organs, leading to tissue pathology [49]. The interactions between immune cells and epithelial/stromal cells during COPD-related inflammation remain poorly understood. In our study, we found that epithelial/stromal cells secrete G-CSF in response to IL-17A produced by γδ^+^ T cells in an emphysema model, driving the differentiation of pathogenic Siglec-F^+^ neutrophils. These findings suggest that epithelial/stromal cells, which are typically involved in host defense, can exacerbate tissue damage by promoting the differentiation of pathogenic immune cells. This finding underscores the critical interactions between epithelial/stromal cells and immune cells within specific pathophysiological environments. Furthermore, epithelial/stromal cells, rather than immune cells, are potential therapeutic targets for diseases such as emphysema. Given that G-CSF is reportedly elevated in the sputum, serum, and BALF of COPD patients [18–20], our findings suggest that G-CSF plays a key role in the differentiation of pathogenic Siglec-F^+^ neutrophils and could serve as a promising therapeutic target for patients with emphysema.

Recent studies have reported that Siglec-F^+^ neutrophils are generated in various inflammatory environments and contribute to the exacerbation of disease symptoms. However, the mechanisms regulating the differentiation of Siglec-F^+^ neutrophils remain unclear. In a mouse model exposed to diesel exhaust particles (DEPs), Siglec-F^+^ neutrophils were shown to enhance the Th2 response, worsening allergic asthma symptoms [27]. Similarly, in a mouse model of kidney injury, Siglec-F^+^ neutrophils promoted the deposition of type 1 collagen, leading to increased renal fibrosis [26]. These reports suggest that Siglec-F^+^ neutrophils may play a consistent role in exacerbating disease symptoms across inflammatory conditions. However, the differentiation of Siglec-F^+^ neutrophils appears to be driven by different cytokines in different disease models. In the DEP model, Siglec-F^+^ neutrophils are observed in the lungs, as in the emphysema model, but their differentiation is triggered by the danger signal ATP [27]. In contrast, in a kidney injury model, GM-CSF and TGF-β drive the differentiation of Siglec-F^+^ neutrophils [26]. In the present study, we also considered GM-CSF as a potential driver of Siglec-F^+^ neutrophil differentiation. However, neutralizing GM-CSF in BM-neutrophil cultures in the presence of IL-17A^WT^-PPE-BALF did not inhibit Siglec-F^+^ neutrophil differentiation. Additionally, inhibiting the phosphorylation of STAT5, a major downstream signal of GM-CSF, failed to suppress G-CSF-mediated Siglec-F^+^ neutrophil differentiation. These results suggest that the Siglec-F^+^ neutrophils generated in the PPE-induced emphysema model may represent a distinct population with different pathogenic characteristics than the Siglec-F^+^ neutrophils observed in other organs. The regulation of Siglec-F^+^ neutrophil differentiation by different signaling pathways, even within the same organ, indicates that each inflammatory disease may involve specific pathogenic immune cells shaped by its unique inflammatory environment.

In conclusion, we identified a population of pathogenic Siglec-F^+^ neutrophils in the lungs of PPE-induced emphysema mice. We revealed the detailed immunobiological mechanisms underlying the differentiation of these pathogenic Siglec-F^+^ neutrophils, which contribute to the aggravation of disease symptoms. Our findings provide valuable insights into the pathogenic mechanisms of PPE-induced emphysema and pave the way for future therapeutic drug development.

## Materials and methods

### Mice and emphysema model

WT C57BL/6 J mice, aged 10–12 weeks, were purchased from DBL (Korea). IL-17A^-/-^ mice, aged 10–12 weeks, were kindly provided by Professor Yeonseok Chung, Seoul National University, Seoul, Republic of Korea. Rag1^-/-^ (B6.129S7-Rag1tm1Mom/J) mice were purchased from the Jackson Laboratory. All the mice used in the experiments were bred on a C57BL/6 background and maintained in the specific pathogen-free animal facility at Sungkyunkwan University in accordance with institutional and university guidelines for the care and use of laboratory animals. To induce emphysema, the mice were first anesthetized with isoflurane, and then, porcine pancreas elastase (E1250; Sigma‒Aldrich) was intratracheally (*i.t*.) instilled at a concentration of 7.5 U/kg.

### Primary cell harvest

All primary cells used in the experiments were isolated from various organs of CO_2_-euthanized mice and processed as follows.

#### Lungs and hearts

The lungs and hearts were perfused with PBS and minced. The samples were then incubated in RPMI 1640 medium (Gibco) containing 60 U/ml DNase I (Enzynomics) and 86 μg/ml collagenase IV (Sigma‒Aldrich) in a gentleMACS Dissociator (Miltenyi Biotec) for 30 min. The resulting cell suspension was filtered through a 70 μm mesh (SPL) and washed with PBS. The cells were lysed with LCK lysis buffer (Gibco) for 5 min at room temperature (RT) and washed twice with PBS.

#### BALF

A catheter attached to a 1 ml syringe was inserted into the mouse trachea, and PBS was injected to collect the BALF. The cells in the collected medium were washed twice with PBS and analyzed via a FACSCanto II (BD Biosciences).

#### Blood

Blood was collected from the retro-orbital sinus of mice anesthetized with isoflurane. The blood was diluted with PBS, layered onto Ficoll (Life Sciences) at a 1:1 ratio, and centrifuged at *800 × g* for 15 min at 20°C with no brake applied. The interface layer was collected, washed twice with PBS, and analyzed by flow cytometry.

#### Bone marrow

Femurs and tibias were isolated from the mice, and bone marrow cells were collected by flushing with PBS through both ends via a syringe. The collected cells were centrifuged to form a pellet, incubated with LCK lysis buffer for 5 min at RT, filtered through 70 μm mesh, washed twice with PBS, and then subjected to flow cytometry and sorting.

#### Spleen

The spleen was ground and passed through 70 μm mesh. The cells were then centrifuged to form a pellet, incubated with LCK lysis buffer for 5 min at RT, washed twice with PBS, and analyzed by flow cytometry.

#### Kidney

The kidneys were perfused with PBS, minced, and incubated in RPMI medium containing 60 U/ml DNase I and 86 μg/ml collagenase IV in a gentleMACS Dissociator for 30 min. The resulting suspension was centrifuged to form a pellet, resuspended in PBS, layered onto a 45–75% Percoll (Sigma–Aldrich) gradient, and centrifuged at *1500 × g* for 20 min. The interface layer was collected, washed twice with PBS, and analyzed by flow cytometry.

#### Blood

Blood was collected from the retro-orbital sinus of isoflurane-anesthetized mice. The blood was diluted with PBS, layered at a 1:1 ratio over Ficoll (Life Sciences) and then centrifuged at *800 × g* for 15 min at 20°C with no brake. The interface layer was collected and washed twice with PBS before flow cytometry analysis.

### Antigen uptake assay

To examine the antigen uptake capacity of neutrophils, the mice were instilled with PPE. Four days later, the lungs were harvested and processed into single-cell suspensions in complete RPMI (cRPMI; RPMI containing 3% fetal bovine serum and 100 U/ml penicillin/streptomycin; Gibco). Subsequently, 1.0 × 10^6^ cells were incubated with 1 μg/ml OVA-AF647 (Invitrogen) at 37°C for 3 h, followed by two washes with PBS. Flow cytometry analysis was then performed.

### NETosis analysis

WT mice were instilled with PPE, and 4 days later, the lungs were harvested and processed into a single-cell suspension using cRPMI. Subsequently, 1.0 × 10^6^ cells were incubated with 1 μM PMA (Sigma‒Aldrich) at 37°C for 3 h, followed by two PBS washes. The cells were then resuspended in FACS flow buffer (BD Biosciences), incubated with 10 nM SYTOX Green (Invitrogen) at 4°C for 15 min, and washed twice with FACS flow buffer. Flow cytometry analysis was then performed.

### Intravascular staining

To assess whether Siglec-F^+^ neutrophils reside in lung tissue, emphysema-induced mice were intravenously injected with 3 µg of the CD45.2-PE mAb. Five minutes later, the lungs were obtained from euthanized mice, perfused with PBS, and then further stained with the anti-CD45.2-PerCPcy5.5 mAb. Flow cytometry analysis was then performed.

### Adoptive transfer of immune cells

To examine the pathogenicity of Siglec-F^+^ neutrophils, WT mice were instilled with PPE. After 48 h, conventional neutrophils (CD45.2^+^Ly6G^+^CD11b^+^Siglec-F^-^) and Siglec-F^+^ neutrophils (CD45.2^+^Ly6G^+^CD11b^+^Siglec-F^+^) were sorted via a FACSAria Fusion system (BD). Subsequently, 1.0 × 10^6^ cells were *i.t*. instilled into naïve WT mice. Two days later, the lungs were perfused and subjected to histological analysis.

### Histology

The lungs were perfused with PBS, fixed in 4% paraformaldehyde (Sigma‒Aldrich), and embedded in paraffin blocks. The paraffin blocks were sectioned to a thickness of 4 μm and subjected to H&E staining. The emphysema index was determined by measuring the MLI. Five random fields at 100× magnification were captured for each sample, and the MLI was calculated via ImageJ software (National Institutes of Health, Maryland, USA; https://imagej.nih.gov/ij/) by overlaying a 50×50 μm grid and dividing the total line length by the number of intersections with alveolar walls.

### ELISA

ELISA was performed as described previously [50], with slight modifications. The lungs were perfused with PBS, homogenized in PBS and subjected to freezing and thawing in liquid nitrogen. Centrifugation was then performed, and the supernatant was collected. Cytokine concentrations were measured via the ELISA MAXTM Deluxe Set Mouse IL-17A, GM-CSF (BioLegend), and Mouse G-CSF DuoSet ELISA (R&D Systems™). In PPE-instilled mice, conventional neutrophils and Siglec-F^+^ neutrophils were sorted from the lungs, resuspended in cRPMI, and seeded at 2.0×10^5^ cells per well in a 96-well plate. After 24 h of restimulation with LPS (1 μg/ml), the supernatants were harvested for cytokine concentration measurements via the ELISA MAXTM Deluxe Set for Mouse TNF-α, IL-6, and IL-10 (BioLegend) and the Mouse IL-1β ELISA Kit (eBioscience).

### In vivo blockade

To neutralize IL-17A and IL-17F in vivo, a group of mice was *i.t*. administered 100 μg of anti-IL-17A (BioXcell), and a separate group was *i.t*. administered 100 μg of anti-IL-17F (BioXcell) one day prior to PPE instillation and on days 1 through 3 postinstillation. A third group of mice was *i.t*. administered 100 μg of both anti-IL-17A and anti-IL-17F simultaneously in the same manner. To neutralize CXCL1 in vivo, the mice were *i.t*. administered 10 μg of anti-CXCL1 (Invitrogen) one day prior to PPE instillation and on days 1 through 3 postinstillation. To neutralize IL-1α, IL-1β, IL-18, and IL-23 in vivo, the mice were *i.t*. administered 80 μg of anti-IL-1α, anti-IL-1β, anti-IL-18 (BioXcell), or anti-IL-23 (Invitrogen) one day prior to PPE instillation and on day 1 postinstillation. For the deletion of Siglec-F^+^ neutrophils, the mice were *i.t*. administered 80 μg of anti-Ly6G (BioXcell) and 40 μg of anti-Siglec-F (R&D Systems) daily for 3 days, starting from the day after PPE instillation. For depletion of γδ^+^ T cells, the mice were *i.t*. administered 80 μg of anti-Vγ2 TCR (BioXcell) one day prior to PPE instillation and on days 1 through 3 postinstillation. For depletion of alveolar macrophages, the mice were *i.t*. administered 30 μl of clodronate (FormuMax Scientific) on the day prior to and the day following PPE instillation. In vivo administration of the inhibitor was performed as described previously [27] with minor modifications. To block the differentiation of Siglec-F^+^ neutrophils, the mice were *i.t*. administered 30 μg of AG490, 20 μg of HJC0152, 3 μg of rapamycin, or 37 μg of adzmapimod (MedChem Express) daily for 3 days, starting from the day after PPE instillation.

### Administration of recombinant IL-17A

Starting the day after PPE instillation in IL-17A^KO^ mice, 1 μg of recombinant IL-17A (PeproTech) was *i.t*. administered daily for 4 days.

### Flow cytometry and sorting

Flow cytometry analysis and cell sorting were performed as described previously [51] with minor modifications. The following antibodies were used in the experiment:

Ly6G-FITC or -APC (clone 1A8), CD45.2-PE (clone 104), CD45.2-PerCPcy5.5 (clone 104), CD3-FITC (clone 17 A2), CD8α-PEcy7 (clone 53-6.7), CD11b-FITC (clone M1/70), CD16-FITC (clone S17014E), TCR γ/δ-PE (clone GL3), Siglec-E-PE (clone M1304A01), CD11b-Pacific Blue (clone M1/70), F4/80-PE (clone BM8), GP38-PE (clone 8.1.1), CD31-APC (clone 390), and B220-FITC (clone RA3-6B2) were purchased from Biolegend. CCR3-PE (clone 83101) and Siglec-F-APCcy7 (clone E50-2440) were purchased from BD Biosciences. CD127-PE (clone SB/199), CD4-eFluor™ 450 (clone GK1.5), CD326-eFluor™ 450 (clone G8.8), IL-17A-APC (clone eBio17B7), and Fixable Viability Dye eFluor™ 506 were purchased from eBioscience.

For neutrophil and eosinophil staining, anti-Ly6G-FITC or anti-APC, anti-CD45.2-PerCPcy5.5 or PEcy7 or PE, anti-CD11b-PE or anti-Pacific blue, anti-Siglec-F-APCcy7, anti-Siglec-E-PE, and anti-CCR3-PE antibodies were used. For epithelial/stromal cell staining, anti-CD45.2-PerCPcy5.5, anti-GP38-PE, anti-CD31-APC, and anti-CD326-Pacific Blue antibodies were used. For alveolar macrophage staining, anti-Ly6G-FITC (to exclude Ly6G^+^ cells), anti-CD45.2-PerCP-Cy5.5, anti-F4/80-PE, and anti-Siglec-F-APC-Cy7 antibodies were used. For T-cell staining, anti-CD3-FITC, anti-TCR γ/δ-PE, anti-CD45.2-PerCPcy5.5, anti-CD8α-PEcy7, and anti-CD4-Pacific Blue antibodies were used. For ILC3 staining, lineage-(anti-CD3-FITC, anti-CD11b-FITC, anti-CD16-FITC, anti-B220-FITC, and anti-CD11c-FITC), anti-CD45.2-PerCPcy5.5, anti-CD127-PE, and anti-CD117-Pacific Blue antibodies were used. Single cells were stimulated with a cell activation cocktail (BioLegend) at 37°C for 4 h. After stimulation, the cells were stained with anti-IL-17A-APC according to the protocol of the Fixation/Permeabilization Kit (BD Biosciences).

### In vitro cell culture

Neutrophils and eosinophils were sorted from the bone marrow and lungs of WT mice via FACSAria Fusion and labeled BM-neutrophils and lung-neutrophils (FVD^-^CD45.2^+^Ly6G^+^CD11b^+^Siglec-F^-^) and BM-eosinophils (FVD^-^CD45.2^+^Ly6G^-^CD11b^+^Siglec-F^+^). BALF was harvested from PPE-instilled WT and IL-17A KO mice via the use of 1 ml of cRPMI. Each BALF sample (100 μl) was used for culturing 4.0 × 10^5^ WT BM-neutrophils in cRPMI for 48 h. In some cases, the cultures of WT BM-neutrophils (4.0 × 10^5^) were supplemented with IL-17A (100 ng/ml), IL-1β (20 ng/ml), IL-6 (20 ng/ml), TNF-α (20 ng/ml) (Peprotech), or GM-CSF (2 ng/ml) (Creagen). In some cases, BM-neutrophils and lung-neutrophils (4.0 × 10^5^ cells each) were cultured in cRPMI supplemented with G-CSF (10 ng/ml) for 48 h. BM-eosinophils (4.0 × 10^5^) were cultured in cRPMI supplemented with either 100 µl of BALF obtained from PPE-instilled WT mice or G-CSF (10 ng/ml) for 48 h. BM-neutrophils (4.0 × 10^5^) were cultured in cRPMI supplemented with 100 µl of cell-free BALF obtained from PPE-instilled WT mice, along with anti-IgG (10 µg/ml), anti-G-CSF (1 μg/ml) (R&D Systems), or anti-GM-CSF (10 µg/ml) (BioXcell), for 48 h.

For the coculture experiments, 4.0 × 10^5^ BM-neutrophils were cocultured with 1.0 × 10^6^ nonimmune (FVD^-^CD45.2^-^) cells sorted from the perfused lungs of WT mice in cRPMI containing IL-17A (100 ng/ml) for 48 h. To further analyze nonimmune cells, 1.0 × 10^6^ FVD^-^CD45.2^-^EpCAM^+^ (epithelial cells), CD31^+^ (blood endothelial cells), CD31^+^GP38^+^ (lymphatic endothelial cells), and GP38^+^ (fibroblasts) cells sorted from the perfused lungs of WT mice were cocultured with 4.0 × 10^5^ BM-neutrophils in cRPMI supplemented with IL-17A (100 ng/ml) for 48 h.

To identify the signaling pathways downstream of G-CSF involved in the differentiation of Siglec-F^+^ neutrophils, 4.0 × 10^5^ BM neutrophils were cultured in cRPMI supplemented with G-CSF (10 ng/ml) in the presence of the following signaling inhibitors: AG490 (50 μM), rapamycin (1 μM), adezmapimod (10 μM), wortmannin (2 μM), STAT5-IN-1 (10 μM) (MedChem Express), and HJC0152 (10 μM) (Selleck) for 48 h.

### RNA isolation

CD45^-^ non-immune cells sorted from the perfused lungs of WT mice were resuspended in cRPMI at 1.0 × 10^6^ cells per well and incubated for 6 h in the presence of IL-17A (100 ng/ml). The cells were lysed via RNAiso Plus (Takara) and purified via the PicoPure™ RNA Isolation Kit (Thermo). For bulk RNA-seq of Siglec-F^+^ neutrophils, BM-neutrophils were sorted from WT mice, seeded at 4.0 × 10^5^ cells per well, and stimulated with G-CSF (10 ng/ml) for 6 h. After washing with PBS, the cells were lysed via RNAiso and purified via the PicoPure™ RNA Isolation Kit.

### Bulk RNA sequencing and analysis

Bulk RNA-seq was performed via a previously [51] described method with slight modifications. The RNA library construction was conducted via a QuantSeq 3′ mRNA-Seq Library Prep Kit FWD (Lexogen) following the manufacturer’s guidelines. The Illumina RNA template containing the compatible sequence was purified via magnetic beads and amplified to add the complete adapter sequences for cluster generation. The sequencing was performed via a high-throughput approach with single-end 75 bp sequencing via a NextSeq 500/550 (Illumina). A volcano plot analysis of upregulated genes in Siglec-F^+^ neutrophils was based on searches conducted at https://maayanlab.cloud/Enrichr/and represents the provided ontology. GSEA was performed via GSEA v4.3.3 software. The gene sets needed for the GSEA were obtained from the murine transcriptomics database provided by the Gene Set Knowledgebase (http://ge-lab.org/gs/). The enrichment score and false discovery rate values were applied after the gene set permutations were performed 1000 times. Raw data from the bulk RNA-seq analysis were deposited in the NCBI Gene Expression Omnibus (GEO) under accession number GSE297403.

### RT‒PCR

The isolated mRNA was used to synthesize cDNA via Maxime™ RT PreMix (Oligo (dT)15 Primer) (iNtRON Biotechnology) following the manufacturer’s protocol. Real-time quantitative PCR was performed using qGreen Q-PCR Master Mix (GenDEPOT). The sequences of primers used for PCR amplification were as follows:

Mouse *Gapdh*

(F) TGA TGG GTG TGA ACC ACG AG

(R) AGT GAT GGC ATG GAC TGT GG

Mouse *Csf3*

(F) TGC ACT ATG GTC AGG ACG AG

(R) TGC TCC AGG GAC TTA AGC AG

### Statistical analysis

Statistical significance was determined via Prism 10 software (GraphPad). Unpaired t tests with Welch’s correction, one-way ANOVA with Dunnett’s T3 post hoc test, or two-way ANOVA with Tukey’s post hoc test were used to evaluate the significance of differences between multiple groups. The data are the means ± SDs.

## Supplementary information

Supplemental Materials

Un-croped images (original)
