# Supplementary material for: γδ+ T-cell-derived IL-17A stimulates airway epithelial/stromal cells to secrete G-CSF, promoting lung-specific pathogenic Siglec-F+ neutrophil development in PPE-induced emphysema
Source: Cell Mol Immunol. 2025 Jun 3;22(7):791–805. doi: 10.1038/s41423-025-01301-x (PMC12206919; doi:10.1038/s41423-025-01301-x)
Supplement: Supplementary file 1 — Supplemental Materials [file 41423_2025_1301_MOESM1_ESM.pdf]

Supplemental Figures

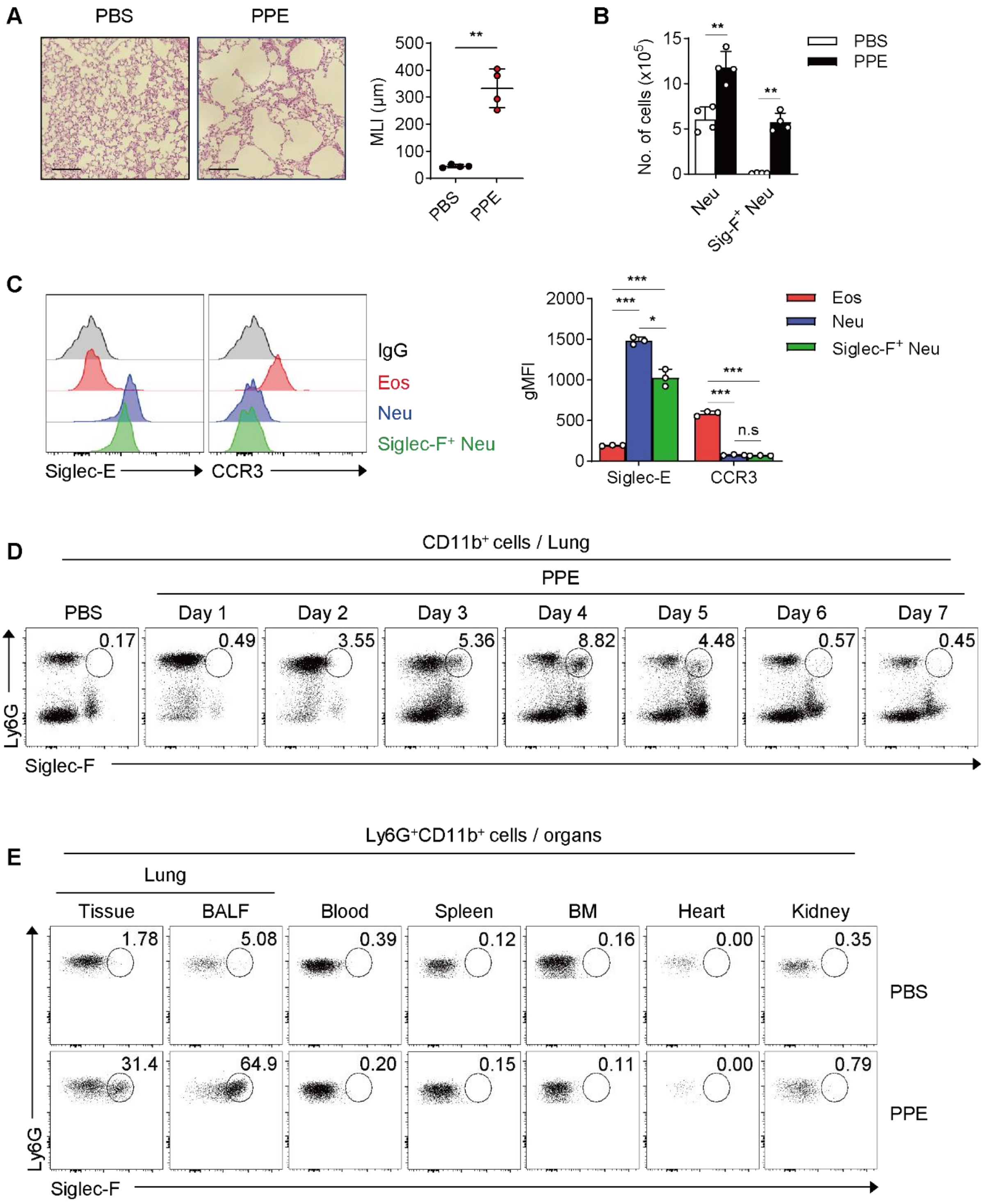

Fig. S1. Siglec-F^{+} neutrophils were newly observed in the lungs of mice with PPE-induced emphysema.

(A) Lung tissue histology after H&E staining and mean linear intercept (MLI) assessment were conducted 4 days after PPE instillation in mice. Scale bar = 200 μm (*n* = 4 per group). (B) Quantification

of neutrophils and Siglec-F$^+$ neutrophils in the lungs of WT mice 4 days after PBS or PPE instillation (*n* = 4 per group). (C) The expression of Siglec-E and CCR3, along with their geometric mean fluorescence intensity (gMFI), in lung immune cells of PPE-instilled mice (*n* = 3 per group). (D) Representative FACS plots of Fig. 1B. (E) Representative FACS plots of Fig. 1C. Unpaired two-tailed Student's t-test with Welch's correction (A), unpaired one-way ANOVA with the Dunnett's T3 post-test (C), unpaired two-way ANOVA with Tukey's for post-test (B) were used to measure significance. *$P < 0.05$, **$P < 0.01$, ***$P < 0.001$; n.s, not significant; error bars indicate mean ± SD.

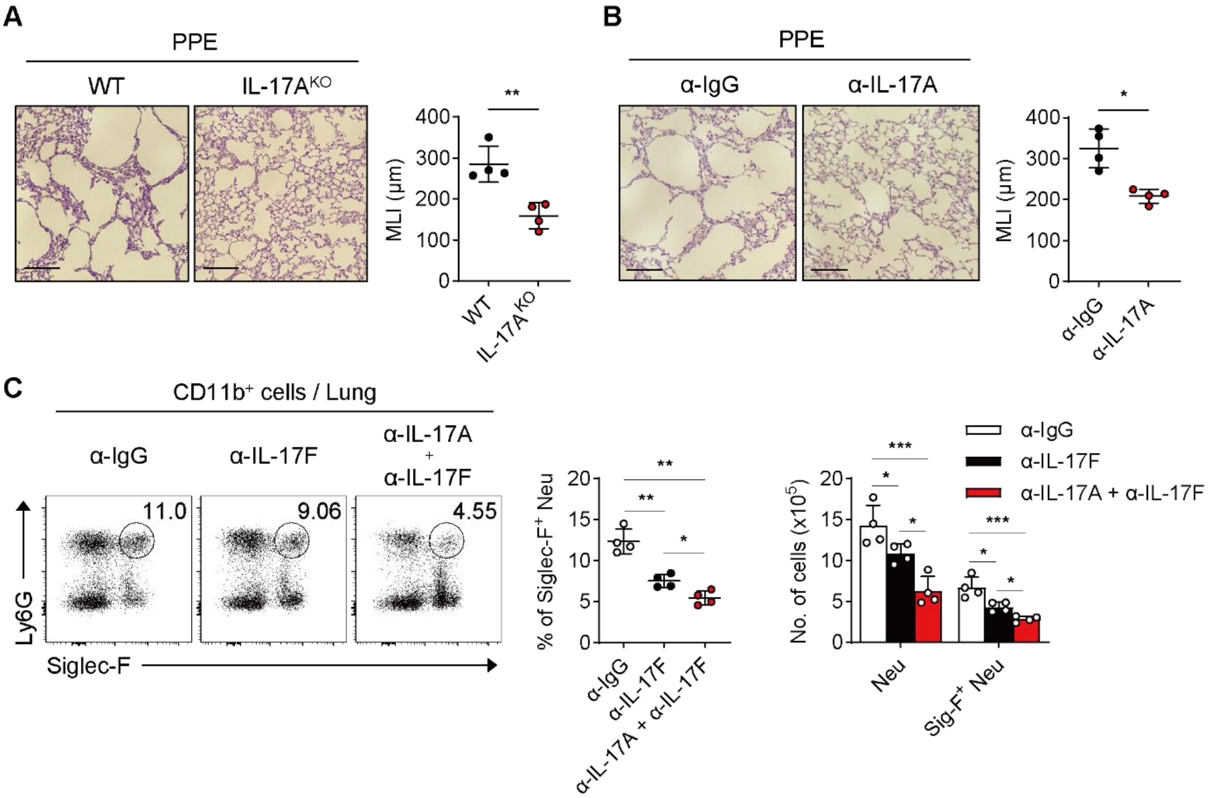

Fig. S2. IL-17A and the development of Siglec-F$^+$ neutrophils in PPE-induced emphysema mice.

(A) Lung tissue histology and the MLI of PPE-instilled WT and IL-17A^{KO} mice on day 4. Scale bar = 200 μm (*n* = 4 per group). (B) Lung tissue histology and the MLI of PPE-instilled WT mice pretreated with either anti-IgG or anti-IL-17A antibodies. Scale bar = 200 μm (*n* = 4 per group). (C) Representative FACS plot of the CD11b$^+$ cell gate and the frequency and number of Siglec-F$^+$ neutrophils in the lungs of PPE-induced emphysema mice following depletion of IL-17A and IL-17F (*n* = 4 per group). Unpaired two-tailed Student's t-test with Welch's correction (A, B), unpaired one-way ANOVA with the Dunnett's T3 post-test (C left), and unpaired two-way ANOVA with Tukey's for post-test (C right) were used to measure significance. *$P < 0.05$, **$P < 0.01$, ***$P < 0.001$; error bars indicate mean ± SD.

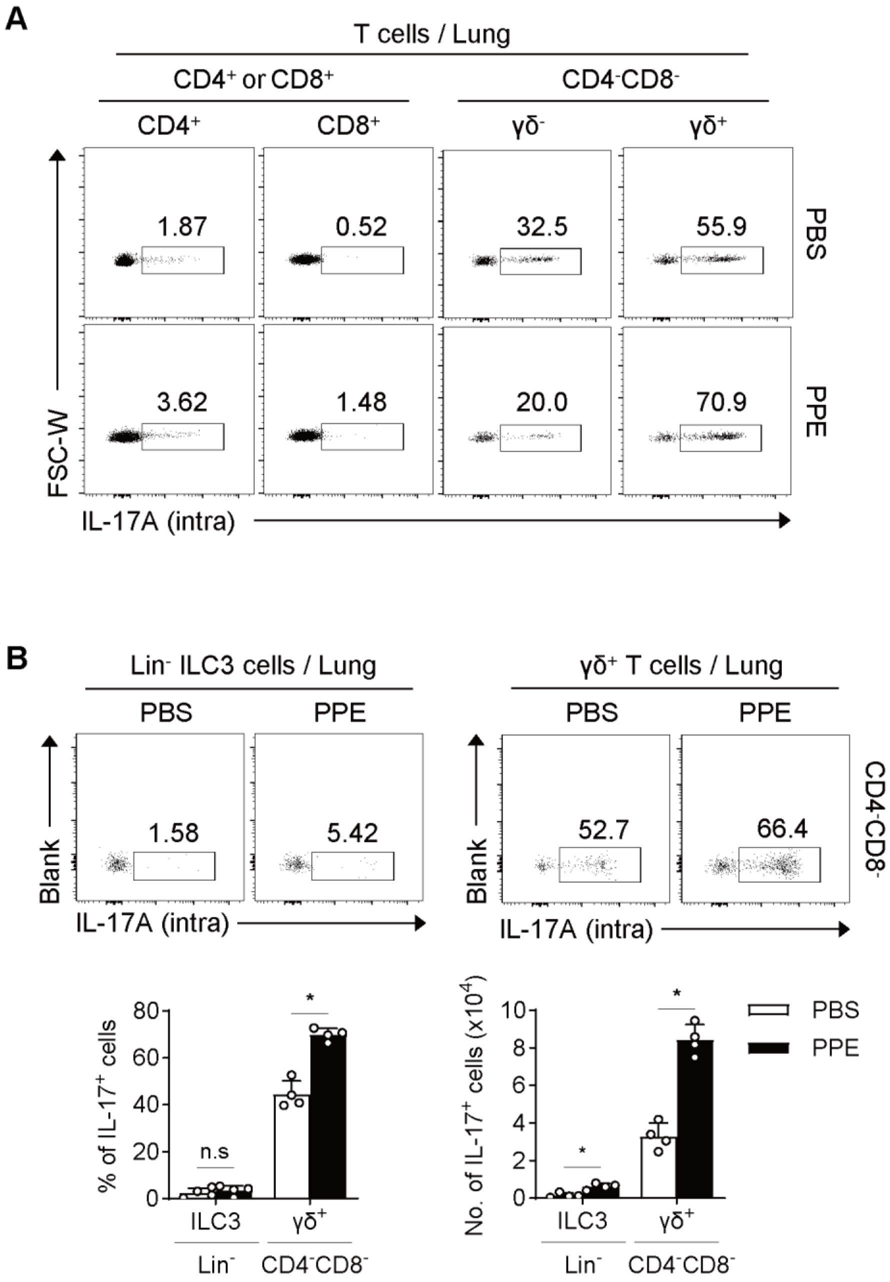

Fig. S3. Lung γδ⁺ T cells are the major source of IL-17A.

(A) Representative FACS plots of main Fig. 3B. (B) The frequency and number of IL-17A-expressing ILC3s and $\gamma\delta^+$ T cells were assessed in the lungs of PPE-instilled mice on day 2 (*n* = 4 per group). Unpaired two-tailed Student's t-test with Welch's correction (B) were used to measure significance. **P* < 0.05; n.s, not significant; error bars indicate mean ± SD.

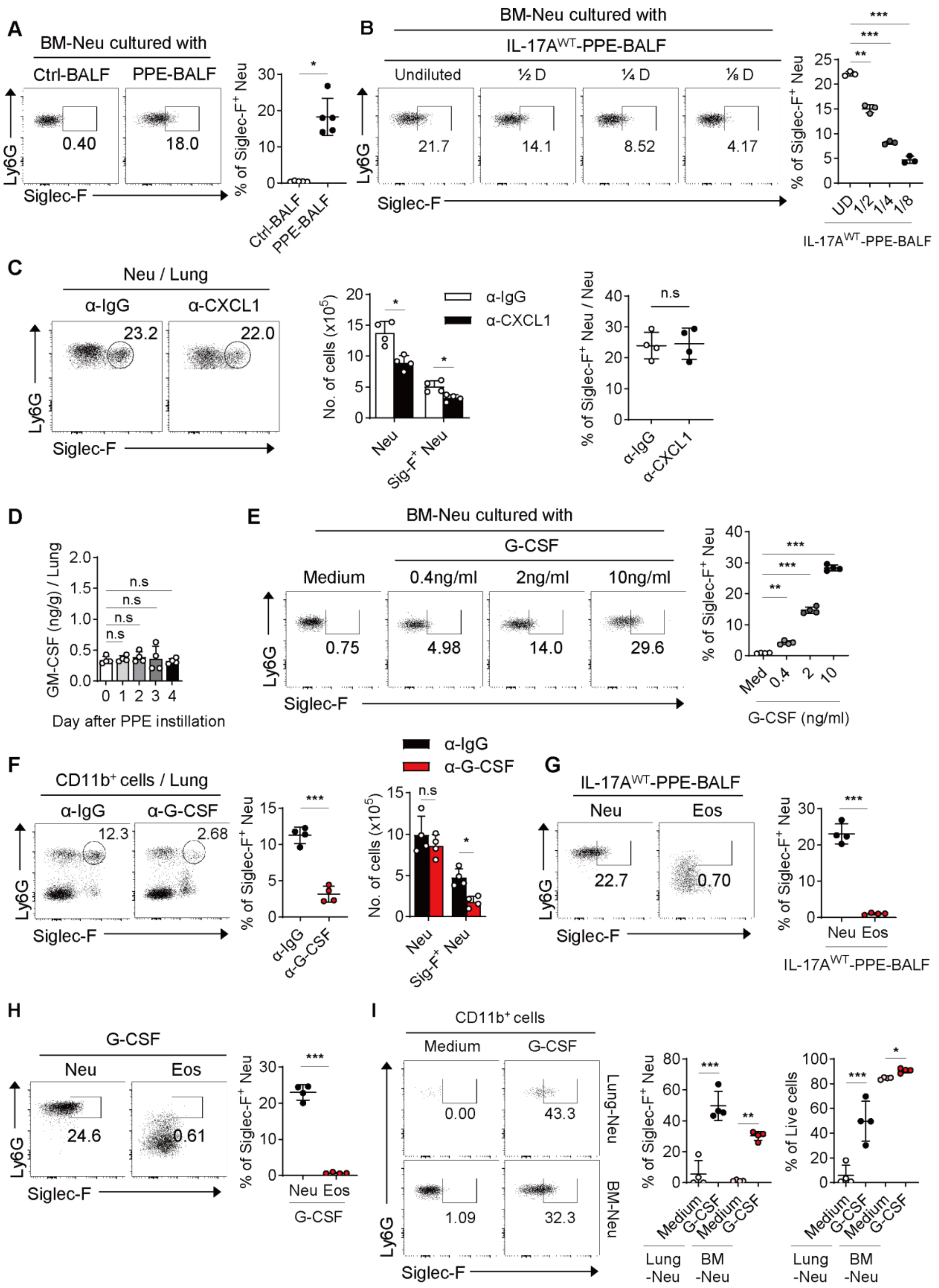

Fig. S4. IL-17A-derived G-CSF plays a crucial and direct role in the differentiation of Siglec-F^{+} neutrophils in the lungs of mice with emphysema.

(A) The frequency of Siglec-F^+ neutrophils in BM-neutrophils cultured for 48 h with cell-free BALF isolated from either PBS- or PPE-instilled WT mice (n = 5 per group). (B) The frequency of Siglec-F^+ neutrophils in BM-neutrophils cultured with serially diluted cell-free BALF isolated from PPE-instilled WT mice (n = 3 per group). (C) Representative FACS plot of the $CD11b^+$ cell gate and the frequency and number of Siglec-F^+ neutrophils in the lungs of PPE-induced emphysema mice following administration of an anti-CXCL1 antibody (n = 4 per group). (D) The concentrations of GM-CSF in the lungs of mice over 4 days following PPE instillation (n = 4 per group). (E) The frequency of Siglec-F^+ neutrophils in BM-neutrophils cultured for 48 h with dose-escalated G-CSF (n = 4 per group). (F) The frequency and number of Siglec-F^+ neutrophils on day 4 in the lungs of PPE-instilled mice pretreated *i.t.* with anti-G-CSF blocking antibodies (n = 4 per group). (G) **N**eutrophils and eosinophils sorted from $CD11b^+$ BM cells were cultured for 48 h with cell-free BALF isolated from PPE-instilled WT mice, and the frequency of Siglec-F^+ neutrophils was assessed in each culture (n = 4 per group). (H) Neutrophils and eosinophils sorted from $CD11b^+$ BM cells were cultured for 48 h with 10 ng/ml of G-CSF, and the frequency of Siglec-F^+ neutrophils was assessed in each culture (n = 4 per group). (I) Neutrophils sorted from lung tissue (Lung-neutrophils) and bone marrow (BM-neutrophils) were cultured with 10 ng/ml of G-CSF, and the frequency of Siglec-F^+ neutrophils was assessed in each culture (n = 4 per group). Unpaired two-tailed Student's t-test with Welch's correction (A, C right, F left, G, and H), unpaired one-way ANOVA with the Dunnett's T3 post-test (B, D, and E), and unpaired two-way ANOVA with Tukey's for post-test (C left, F right, and I) were used to measure significance. *$P < 0.05$, **$P < 0.01$, ***$P < 0.001$; n.s, not significant; error bars indicate mean ± SD.

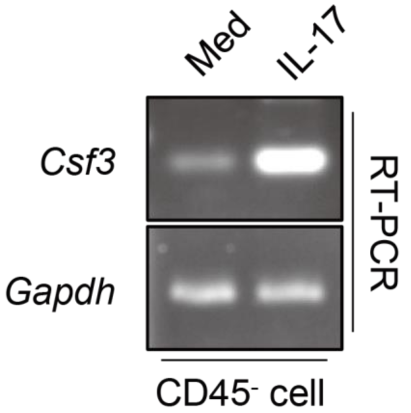

Fig. S5. Lung non-immune cells secrete G-CSF when stimulated by IL-17A.

The expression levels of *Csf3* mRNA were assessed by RT-PCR in CD45$^-$ cells treated with recombinant IL-17A for 6 h. Representative RT-PCR data from three independent experiments.

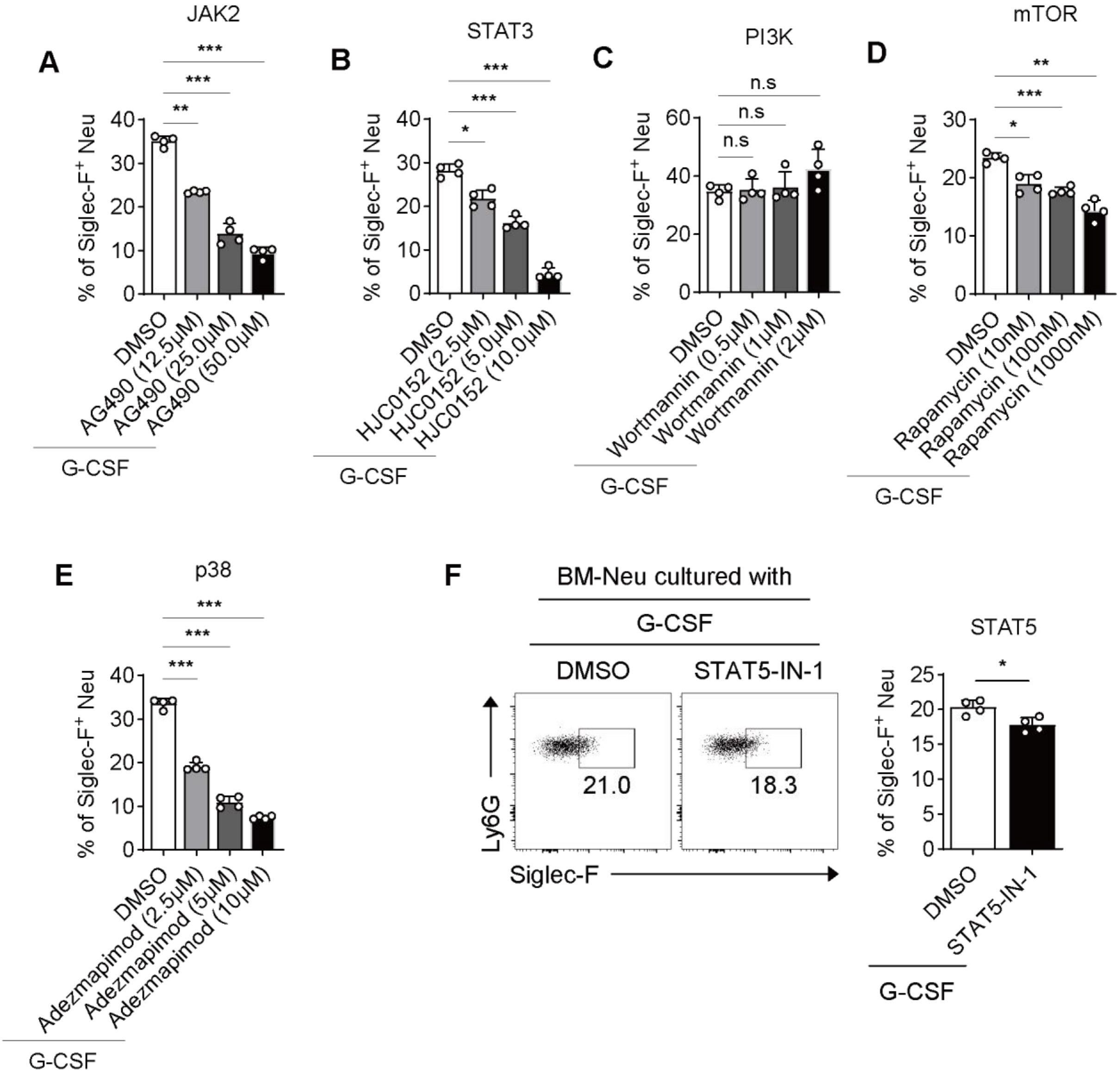

Fig. S6. G-CSF receptor signaling pathways for the expression of Siglec-F in neutrophils.

(A-E) The frequency of Siglec-F$^+$ neutrophils was assessed in each BM-neutrophil culture after 48 h of incubation with 10 ng/ml of recombinant G-CSF, in the presence of dose-escalating AG490 (JAK2 inhibitor) (A), HJC0152 (STAT3 inhibitor) (B), Wortmannin (PI3K inhibitor) (C), Adezmapimod (p38 inhibitor) (D), or rapamycin (mTOR inhibitor) (E). (F) The frequency of Siglec-F$^+$ neutrophils was assessed in BM-neutrophil culture after 48 h of incubation with 10 ng/ml of recombinant G-CSF, in the presence of STAT5-IN-1 (STAT5 inhibitor). (A-F) (n=4 per group). Unpaired two-tailed Student's t-test with Welch's correction (F) and unpaired one-way ANOVA with the Dunnett's T3 post-test (A-E) were used to measure significance. *P < 0.05, **P < 0.01, ***P < 0.001; n.s, not significant; error bars indicate mean ± SD.

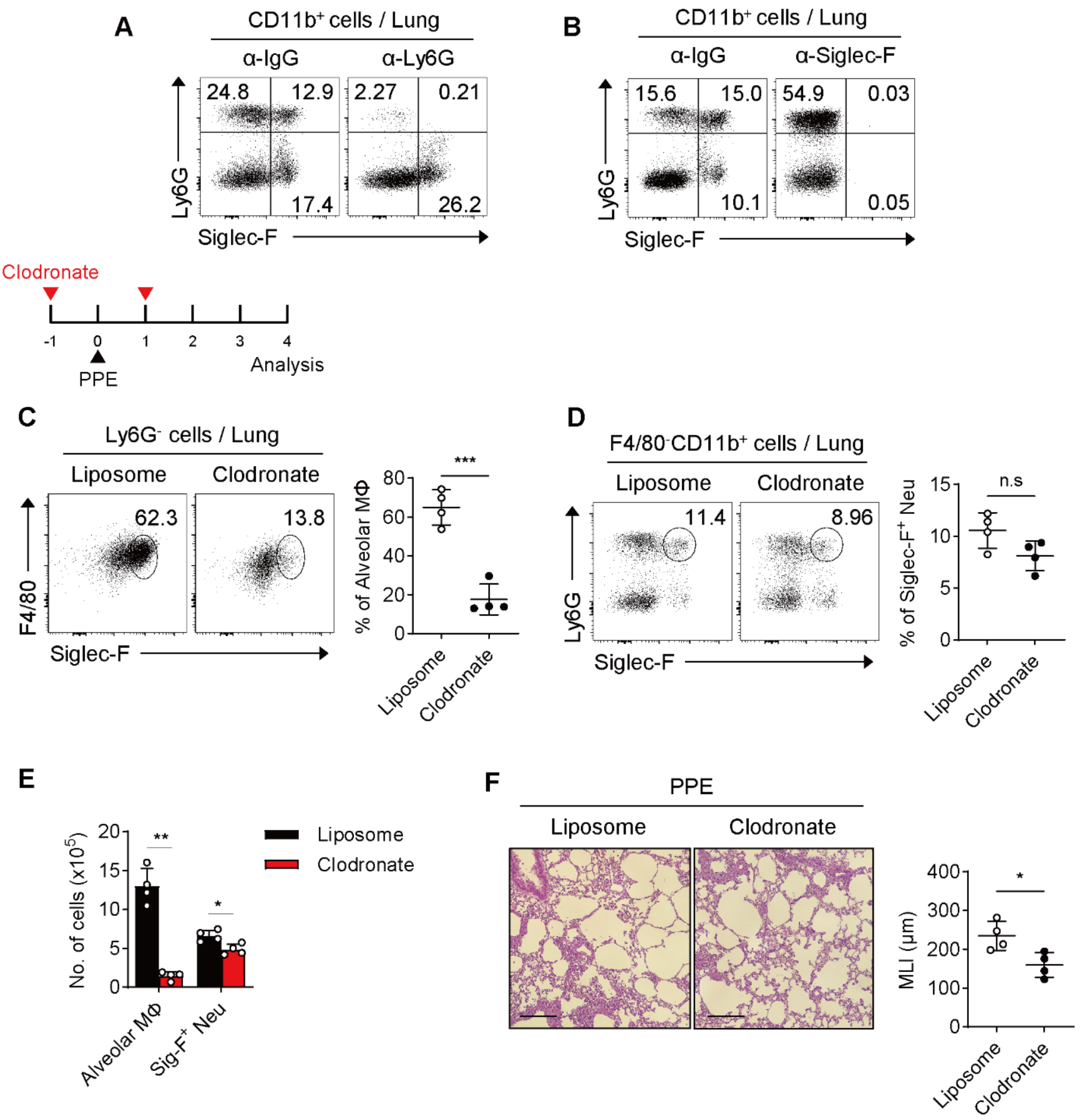

Fig. S7. Depletion of Siglec F^{+} neutrophil and alveolar macrophage in mice with PPE-induced emphysema.

(A, B) Representative FACS plots of the statistical data shown in Fig. 8A (A) and 8B (B). (C-E) Experimental protocol for Clodronate administration in the PPE-induced emphysema model. The frequency of alveolar macrophages (C) and Siglec-F^{+} neutrophils (D), and the number of alveolar macrophages and Siglec-F^{+} neutrophils (E) on day 4 in the lungs of PPE-induced emphysema mice after Clodronate administration (n = 4 per group. (F) Lung tissue histology after H&E staining and MLI assessment on day 4. Scale bar = 200 μm (n = 4 per group). Unpaired two-tailed Student's t-test (C, D, and F) and unpaired two-way ANOVA with Tukey's for post-test (E) were used to measure significance. *P < 0.05, **P < 0.01, ***P < 0.001; error bars indicate mean ± SD.

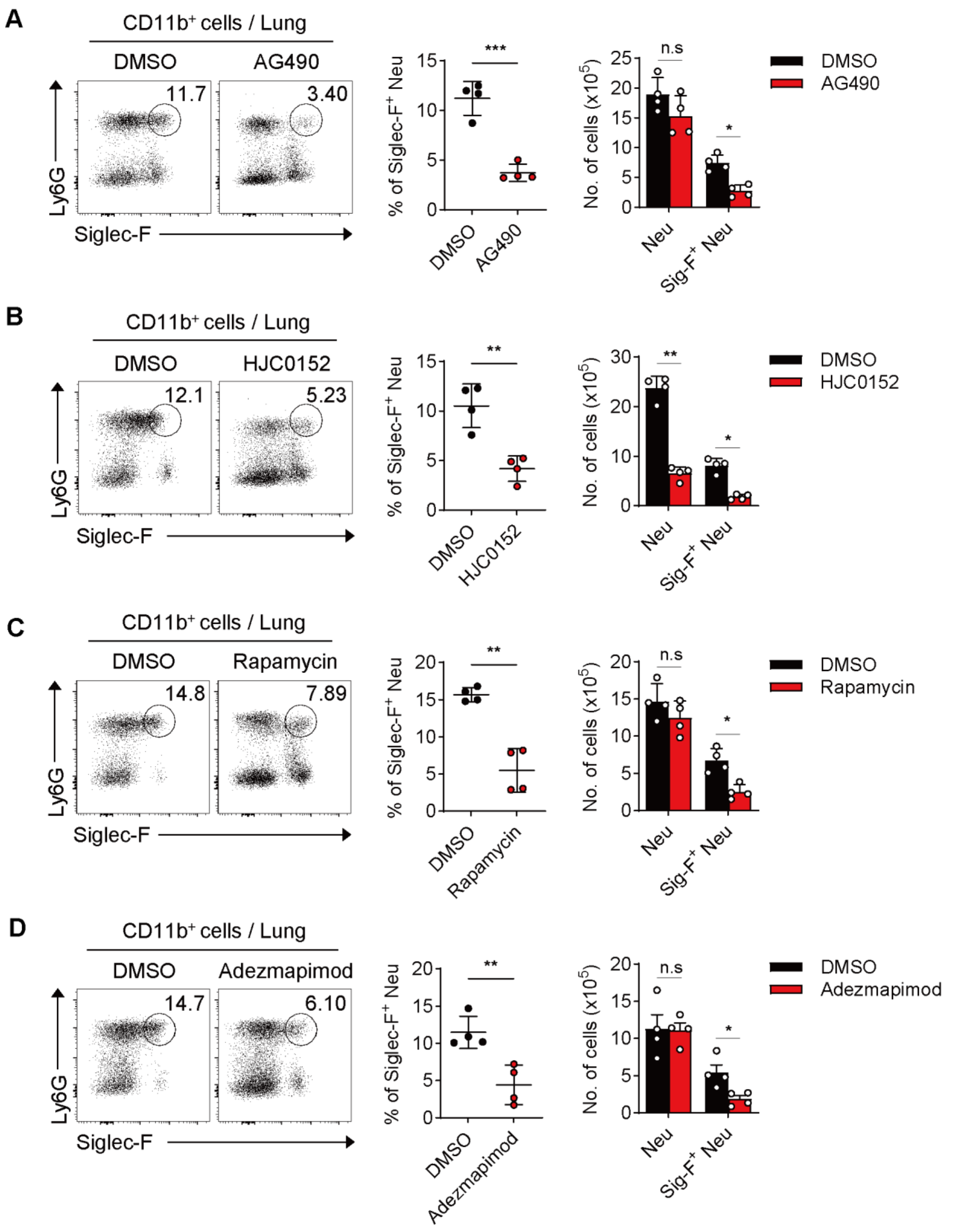

Fig. S8. G-CSF receptor Signaling blockades and symptoms of emphysema.

(A-D) PPE-instilled mice were treated *i.t.* with AG490 (JAK2 inhibitor) (A), HJC0152 (STAT3 inhibitor) (B), Rapamycin (mTOR inhibitor) (C), or Adezmapimod (p38 inhibitor) (D). Representative FACS plots of CD11b^{+} cell gate and the frequency of Siglec-F^{+} neutrophils in the lungs 4 days after PPE

instillation (*n* = 4 per group per each experiment). Unpaired two-tailed Student's t-test (A-D left) and unpaired two-way ANOVA with Tukey's for post-test (A-D right) was used to measure significance. $^{**}P < 0.01$, $^{***}P < 0.001$; error bars indicate mean ± SD.

Supplemental Tables

Supplemental Table 1. Dyes and antibodies for flow cytometry

Reagent	Clone	Source	Catalog #	Diluted ratio
anti-Ly6G-FITC	1A8	Biolegend	127606	1:200
anti-Ly6G-APC	1A8	Biolegend	127613	1:200
CD45.2-PE	104	Biolegend	109807	*In vivo* injection
CD45.2-PerCPcy5.5	104	Biolegend	109827	1:200
CD3-FITC	17A2	Biolegend	100204	1:100
CD8-PEcy7	53-6.7	Biolegend	100721	1:200
CD11b-FITC	M1/70	Biolegend	101205	1:200
CD16-FITC	S17014E	Biolegend	158007	1:200
TCR γ/δ-PE	GL3	Biolegend	118107	1:200
Siglec-E-PE	M1304A01	Biolegend	677103	1:200
GP38-PE	8.1.1	Biolegend	127407	1:200
CD31-APC	390	Biolegend	102409	1:200
B220-FITC	RA3-6B2	Biolegend	103205	1:200
F4/80-PE	BM8	Biolegend	123110	1:200
CD11b-Pacific Blue	M1/70	Biolegend	101223	1:200
CCR3-PE	83101	BD Bioscience	FAB729P	1:200
Siglec-F-APCcy7	E50-2440	BD Bioscience	565527	1:200
CD127-PE	SB/199	eBioscience	12-1273	1:200
CD4-eFluor™ 450	GK1.5	eBioscience	48-0041-82	1:200
CD326-eFluor™ 450	G8.8	eBioscience	48-5791-82	1:200
IL-17A-APC	eBio17B7	eBioscience	17-7177-81	1:200
Fixable Viability Dye eFluor™ 506	-	eBioscience	65-0866	1:400

Supplemental Table 2. Chemicals, recombinant proteins and antibodies used in this study

Reagent	Clone	Source	Catalog #
Porcine pancreas elastase	-	Sigma-Aldrich	E1250
AG490	-	MedChem Express	HY-12000
HJC0152	-	Selleck	S8561
Rapamycin	-	MedChem Express	HY-10219
Adezmapimod	-	MedChem Express	HY-10256
Wortmannin	-	MedChem Express	HY-10197
STAT5-IN-I	-	MedChem Express	HY-101853
OVA-AF647	-	Invitrogen	O34784
SYTOX Green	-	Invitrogen	S7020
PMA	-	Sigma-Aldrich	P1585
Cell Activation Cocktail	-	Biolegend	423303
RNAiso Plus	-	Takara	9109
Collagenase IV from *Clostridium histolyticum*	-	Sigma-Aldrich	C5138
RPMI 1640 Medium	-	Gibco	11875119

DNase I	-	Enzynomics	M059S
Fetal Bovine Serum (FBS)	-	Gibco	12483020
Penicillin/Streptomycin	-	Gibco	15140122
ACK lysing buffer	-	Gibco	A1049201
Percoll	-	Sigma-Aldrich	P1644
Ficoll	-	Life Sciences	17544202
LPS from Escherichia coli O111:B4	-	Sigma-Aldrich	L4391
Recombinant mouse IL-17A	-	Peprotech	210-17
Recombinant mouse G-CSF	-	Peprotech	250-05
Recombinant mouse IL-1β	-	Peprotech	211-11B
Recombinant mouse IL-6	-	Peprotech	216-16
Recombinant mouse TNF-α	-	Peprotech	315-01A
Recombinant mouse GM-CSF	-	Creagen	JW-M001
anti-IgG2a	2A3	BioXcell	BE0089
anti-CXCL1	Arg20-Lys96	Invitrogen	MA5-23745
anti-mouse GM-CSF	MP1-22E9	BioXcell	BE0259
anti-normal goat IgG	N/A	R&D systems	AB-108-C
anti-G-CSF	N/A	R&D systems	AF-414-NA
anti-IgG1	MOPC-21	BioXcell	BE0083
anti-IL-17A	17F3	BioXcell	BE0173
anti-IL-17F	MM17F8F5.1A9	BioXcell	BE0303
anti-IgG	N/A	BioXcell	BE0091
anti-Vγ2 TCR	UC3-10A6	BioXcell	BE0168
anti-IL-1α	MOPC-21	BioXcell	BE0243
anti-IL-1β	BE024617F3	BioXcell	BE0246
anti-IL-18	YIGIF74-1G7	BioXcell	BE0237
anti-IL-23	eBio473P19	Invitrogen	14-7238-85

Supplementary Table 3. Assay kits used in this study

Kit	Source	Catalog #
Fixation/Permeabilization Kit	BD Biosciences	554714
Maxime™ RT PreMix (Oligo (dT)15 Primer)	iNtRON Biotechnology	25081
qGreen Q-PCR Master Mix	GenDEPOT	Q5600-005
PicoPure™ RNA Isolation Kit	Thermo	KIT0204
Clophosome®-A - Clodronate Liposomes	FormuMax Scientific	F70101C-AC

Supplementary Table 4. Primers for (q)RT-PCR

Mouse *Csf3* Forward	5' - TGC ACT ATG GTC AGG ACG AG - 3'
Mouse *Csf3* Reverse	5' - TGC TCC AGG GAC TTA AGC AG - 3'
Mouse *Gapdh* Forward	5' - TGA TGG GTG TGA ACC ACG AG - 3'
Mouse *Gapdh* Reverse	5' - AGT GAT GGC ATG GAC TGT GG - 3'
