# Supplementary figures and images for: γδ+ T-cell-derived IL-17A stimulates airway epithelial/stromal cells to secrete G-CSF, promoting lung-specific pathogenic Siglec-F+ neutrophil development in PPE-induced emphysema
Source: Cell Mol Immunol. 2025 Jun 3;22(7):791–805. doi: 10.1038/s41423-025-01301-x (PMC12206919; doi:10.1038/s41423-025-01301-x)

## Slide 1
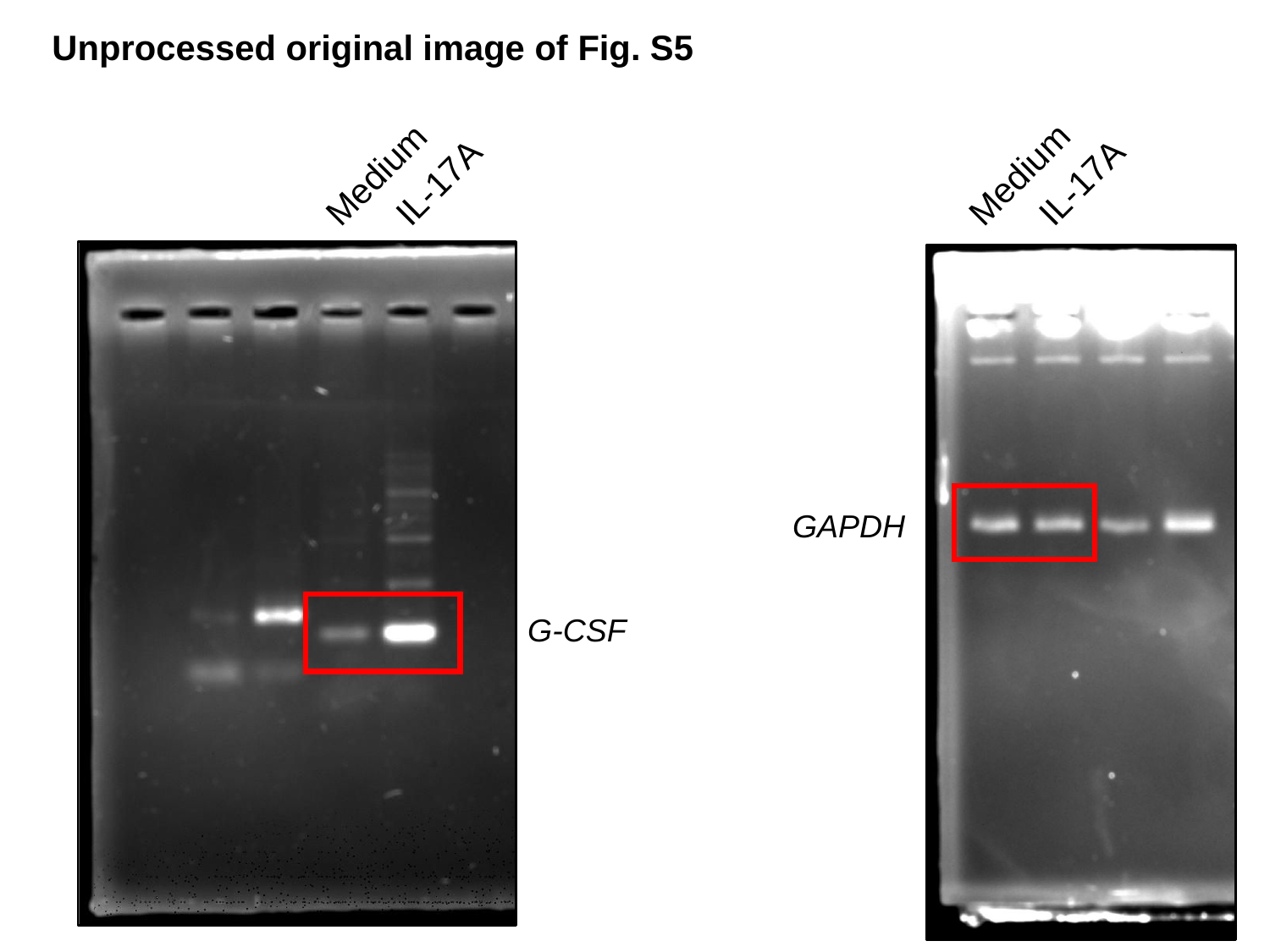

Unprocessed original image of Fig. S5
Medium
IL-17A
Medium
IL-17A
GAPDH
G-CSF

Supplement: Supplementary file 2 — Un-croped images (original) [file 41423_2025_1301_MOESM2_ESM.pptx]
